# Ice Inhibition for Cryopreservation: Materials, Strategies, and Challenges

**DOI:** 10.1002/advs.202002425

**Published:** 2021-02-01

**Authors:** Tie Chang, Gang Zhao

**Affiliations:** ^1^ Department of Electronic Science and Technology University of Science and Technology of China Hefei Anhui 230027 China

**Keywords:** chemical ice‐inhibition molecules, cryopreservation, engineering strategies, external physical field, ice inhibition

## Abstract

Cryopreservation technology has developed into a fundamental and important supporting method for biomedical applications such as cell‐based therapeutics, tissue engineering, assisted reproduction, and vaccine storage. The formation, growth, and recrystallization of ice crystals are the major limitations in cell/tissue/organ cryopreservation, and cause fatal cryoinjury to cryopreserved biological samples. Flourishing anti‐icing materials and strategies can effectively regulate and suppress ice crystals, thus reducing ice damage and promoting cryopreservation efficiency. This review first describes the basic ice cryodamage mechanisms in the cryopreservation process. The recent development of chemical ice‐inhibition molecules, including cryoprotectant, antifreeze protein, synthetic polymer, nanomaterial, and hydrogel, and their applications in cryopreservation are summarized. The advanced engineering strategies, including trehalose delivery, cell encapsulation, and bioinspired structure design for ice inhibition, are further discussed. Furthermore, external physical field technologies used for inhibiting ice crystals in both the cooling and thawing processes are systematically reviewed. Finally, the current challenges and future perspectives in the field of ice inhibition for high‐efficiency cryopreservation are proposed.

## Graphical abstract

This work describes the fundamental mechanisms of ice injury during cryopreservation, and introduces the state‐of‐the‐art ice‐inhibition materials and strategies, both in the cooling and thawing processes, for high‐efficiency cryopreservation. Future perspectives and challenges are also proposed to motivate the development of cell, tissue, and organ cryopreservation and offer bright new inspiration for cryobiology.

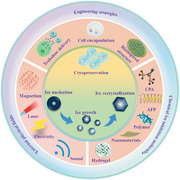

## 1 Introduction

Cryopreservation is a basic and important technique to achieve long‐term storage of organs, tissues, cells, and other biological materials by using a very low temperature (at −80 or −196 °C).^[^ ^1^, ^2^ ^]^ At the cryopreserved temperature condition, the chemical and biological reactions in living cells significantly reduce, and even stop, which is the fundamental mechanism for achieving long‐term preservation of various biological samples.^[^ ^3^, ^4^ ^]^ Cryopreserved cells or tissues not only maintain normal structure and function integrity after thawing from cryogenic temperatures, but also allow for further clinical application and fundamental research.^[^ ^5^ ^]^ Until now, the basic theories, cryogenic devices, cryopreservation strategies, and novel cryoprotectants (CPAs) for cryopreservation have made a breakthrough in the field of cryobiology, thus promoting considerable development of numerous biomedical applications for cryopreservation, including assisted reproduction, stem cell therapies, regenerative medicine, tissue engineering, biological sample banking, and the development and research of drugs.^[^ ^6^, ^7^, ^8^, ^9^ ^]^ For example, with recent advances in flourishing stem cell‐based medicine, the demand for stem cells is sharply increasing.^[^ ^10^, ^11^, ^12^, ^13^ ^]^ Hence, achieving high‐quality and high‐efficiency storage of stem cells is vital to overcome the current supply–demand imbalance.^[^ ^12^, ^14^ ^]^ Additionally, another typical application area is human fertility. Recently, the risk of infertility in young women has been increasing due to occasional disease, aggressive medicine use, and external pressures.^[^ ^15^ ^]^ The reproductive diseases occurring in young men worldwide seriously affect their fertility continuity.^[^ ^16^ ^]^ Therefore, the cryopreservation of egg cells, sperm, ova, and embryos are crucial for human reproduction.^[^ ^17^, ^18^, ^19^ ^]^ In summary, the primary and indispensable cryopreservation science for various biological samples (e.g., cells, tissues, organs, and vaccines) is vital for clinical applications and scientific research in the field of biomedical engineering.^[^ ^20^, ^21^, ^22^, ^23^, ^24^ ^]^

In the process of cryopreservation, a variety of chemical and physical damage occurring in the procedures of freeze–thawing are the main destruction mechanisms of cryopreserved biological samples.^[^ ^25^, ^26^ ^]^ Among these injuries, the formation, growth, and recrystallization of ice crystals are fatal for cryopreserved samples, causing primary problems and limitations for realizing efficient cryopreservation.^[^ ^27^, ^28^ ^]^ It is well known that liquid water is essential for maintaining the structure and function integrity of living cells. However, the inevitable water‐to‐ice phase transition (intracellular and extracellular ice) at the freezing temperature usually leads to cells sustaining mechanical ice damage.^[^ ^29^ ^]^ The elevated solute concentration due to ice formation also results in irreversible injury to cells after undergoing freeze–thaw cycles.^[^ ^30^, ^31^ ^]^ To date, cryopreservation strategies are mainly divided into the slow freezing and vitrification methods.^[^ ^5^ ^]^ For the most common and traditional slow‐freezing techniques, despite allowing low‐CPA solution, ice injury both in intracellular and extracellular solutions is a crucial factor for cell survival.^[^ ^32^, ^33^ ^]^ To minimize ice damage, many cryogenic researchers have put great effort into optimizing the parameters of cryopreserved conditions, including the concentrations of CPA, cooling rates, cooling temperature, and warming rates.^[^ ^34^, ^35^, ^36^ ^]^ Owing to the advantage of ice‐free solution in the process of freezing, the vitrification method has attracted considerable attention, and is regarded as the most promising way to achieve organ cryopreservation in the future.^[^ ^37^ ^]^ However, the main limitation of vitrification is the formation of ice nucleation and devitrification in the warming process, which causes fatal injury to cryopreserved samples.^[^ ^38^, ^39^, ^40^, ^41^ ^]^ It is self‐evident that the formation and growth of ice crystals, as well as the accompanying mechanical damage, are the dominant reasons for the destruction of the function and structure of cryopreserved biological samples. Hence, the control, restriction, and elimination of ice crystals are fundamental and crucial scientific problems in the field of cryopreservation.

**Scheme 1:**
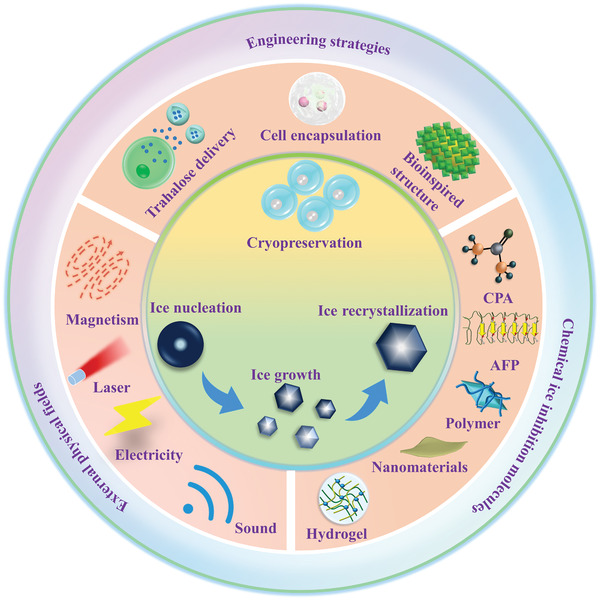
Current emerging ice‐inhibition materials and strategies for cryopreservation.

With the rapid development of biology, chemistry, and material research, innovative materials and biotechnology tools are utilized to suppress the formation and growth of ice crystals; this offers a revolutionary improvement to enhance cryopreservation science.^[^ ^42^, ^43^, ^44^, ^45^ ^]^ Nevertheless, to the best of our knowledge, a systematic and comprehensive review of ice control for cryopreservation has not been found. To this end, as depicted in **Scheme** **1**, the latest developments and progress on materials and strategies to regulate ice crystals for cryopreservation are summarized. First, we introduce the general background and fundamental ice injury mechanisms in the cryopreservation process. Then, we highlight the chemical ice‐inhibition molecules for cryopreservation, including CPAs, antifreeze proteins (AFPs), synthetic polymers, nanomaterials, and hydrogels. Moreover, advanced engineering strategies, including intracellular trehalose delivery, cell encapsulation, bioinspired structure design, and external physical field technologies for ice inhibition in the freezing and warming processes are discussed. Finally, the current challenges and future perspectives on ice regulation are outlined, to motivate the flourishing development of cryopreservation in the field of biomedical engineering. By highlighting the materials and advanced technologies for ice inhibition during cryopreservation, we hope to bring new inspiration and insights into high‐efficiency cryopreservation for biomedical engineering.

## 2 Fundamental Ice Injury during Cryopreservation

**Figure 1:**
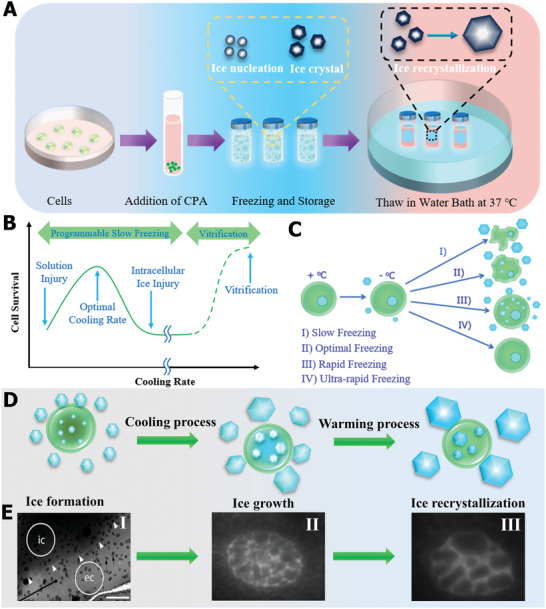
Fundamental ice injury in the process of cryopreservation. A) The basic procedures and cryodamage mechanisms during cryopreservation. B) The dependence of cell rate on cooling rates. C) Cell volume response and injuries correspond to cooling rates. B,C) Reproduced with permission.^[^ Copyright 2017, Elsevier. D) Fundamental ice damage to cells during cryopreservation. E) Typical images of extracellular and intracellular ice. Reproduced with permission.^[^ ^47^ ^]^ Copyright 2019, American Chemical Society. Reproduced with permission.^[^ ^48^ ^]^ Copyright 2016, Elsevier.

As a fundamental and important approach to storing biological specimens, cryopreservation can effectively reduce metabolism and offer vital support for various biological applications. The basic cryopreservation procedures and cryoinjuries that occur during the cooling and warming processes are summarized in **Figure** **1**A. Remarkably, the formation and growth of ice crystals during the freezing and thawing process of cryopreservation is the primary problem that results in the loss of cell viability. Ice crystals are inevitable in the full cryopreservation process, and their control and inhibition is critical to minimizing cellular damage.

Generally, the ice injury can be divided into extracellular and intracellular ice during the freeze–thaw cycle process. It is well known that the survival of cryogenic cells greatly depends on the cooling rates,^[^ ^33^, ^38^ ^]^ which can be defined as slow freezing and vitrification (Figure 1B). As illustrated in Figure 1C, according to the tow‐factor hypothesis theory,^[^ ^32^ ^]^ regarding slow freezing, most intracellular water flows out because the chemical potential of intracellular water is higher than that of the extracellular ice phase, which results in the dehydration of cells, and, thus, causes extracellular ice and osmotic pressure damage for the cell. With the increase in the cooling rate, intracellular water cannot flow out as quickly, thus forming intracellular ice and leading to fatal cryoinjury to cells in the freezing process. In brief, regardless of slow or rapid freezing, ice‐crystal formation is inevitable, and optimizing the extracellular and intracellular ice crystals in the cooling process and minimizing ice injury to cells is crucial. Notably, vitrification cryopreservation can avoid ice injury during the freeze process due to the ice‐free status of the solution with the aid of a high concentration of CPAs. However, the devitrification and recrystallization occurring during the thawing stage can cause fatal damage to cryopreserved cells. In addition, the toxicity of high‐concentration CPA is also a major problem and limitation for the vitrification cryopreservation strategy. In conclusion, ice nucleation formation, ice‐crystal growth, and ice recrystallization/devitrification are the three main factors that restrict cryopreservation efficiency and quality (Figure 1D).

Extracellular ice not only leads to mechanical damage to the cell, but also causes an accompanying increase in solute concentration, resulting in osmotic injury.^[^ ^49^ ^]^ As illustrated in Figure 1E‐I, the cryo‐EM images showed that small hexagonal ice crystals occurred and were distributed outside the biological samples.^[^ ^48^ ^]^ In other words, the survival condition of cells is associated with the status of the extracellular solution that results from ice formation. For the slow‐freeze approach, extensive experimental evidence has proven that the reduction of remaining unfrozen water can cause serious damage to cells.^[^ ^28^, ^48^ ^]^ With respect to the injury mechanism, Mazur and Rigopoulos discovered that the cryogenic cells lay within liquid‐filled channels, and the mass of cells has a great influence on the final cryopreserved efficiency due to the cell‐to‐cell contact effect.^[^ ^50^ ^]^ Moreover, extracellular ice plays an important role in the process of warming. It is well known that the risky temperature zone (from −15 to −160 °C) in the thawing process promotes the transformation of some small ice crystals and liquid water into larger ice crystals.^[^ ^25^ ^]^ Currently, inhibition of the formation and growth of ice crystals during the warming process is of great significance for improving the efficiency of cryopreservation, and remains a research hotspot in the field of cryogenic storage. Overall, ice recrystallization is a complex process, and is closely correlated with different warming parameters. Warming rates and temperatures are vital influencing factors for the nucleation and growth of extracellular ice.

Compared with extracellular ice, intracellular ice occurs in the condition of rapid cooling, as a result of the frozen intracellular water. In order to overcome major intracellular‐injury obstacles for cryopreserved cells and tissues, understanding the underlying intracellular ice‐formation mechanism and controlling intracellular ice via experiment and theory analysis have attracted tremendous attention in the past decades.^[^ ^51^, ^52^, ^53^, ^54^ ^]^ With respect to intracellular ice formation, the main theories that elucidate the intracellular formation mechanisms include pore theory, surface‐catalyzed nucleation (SCN) theory, volume‐catalyzed nucleation (VCN) theory, and cell membrane damage theory.^[^ ^52^, ^55^, ^56^, ^213^ ^]^ In addition to these theories, numerous experimental data have demonstrated that intracellular ice is affected by many factors, such as cooling rates, permeability to water, intracellular supercooling, the integrity of cell membranes, and extracellular ice. Typically, the establishment of a prediction model and equations for intracellular ice, developed by Mazur, demonstrated that the formation of intracellular ice mainly depended on the cooling rates and the permeability of water to cells, which provides insight into the determining factors for cell survival.^[^ ^57^ ^]^ Likewise, in the process of warming, variable parameters influence intracellular ice nucleation and growth. As shown in Figure 1E‐II,III, the number of ice crystals decreased, but the size of individual ice crystals was larger during cryopreservation, indicating the occurrence of recrystallization.^[^ ^47^ ^]^ Furthermore, it has been proved that intracellular ice produced more serious recrystallization under slower warming rates at the same temperature condition, implying that the faster warming rate is helpful in inhibiting recrystallization and weakening intracellular ice injury. Indeed, intracellular ice can result in a fatal injury to cryogenic cells in freeze–thaw cycles. Thus, exploring the formation mechanism of intracellular ice and eliminating accompanying injury to organelles remains a research priority in the field of cryobiology.

## 3 Chemical Ice‐Inhibition Molecules for Cryopreservation

**Figure 2:**
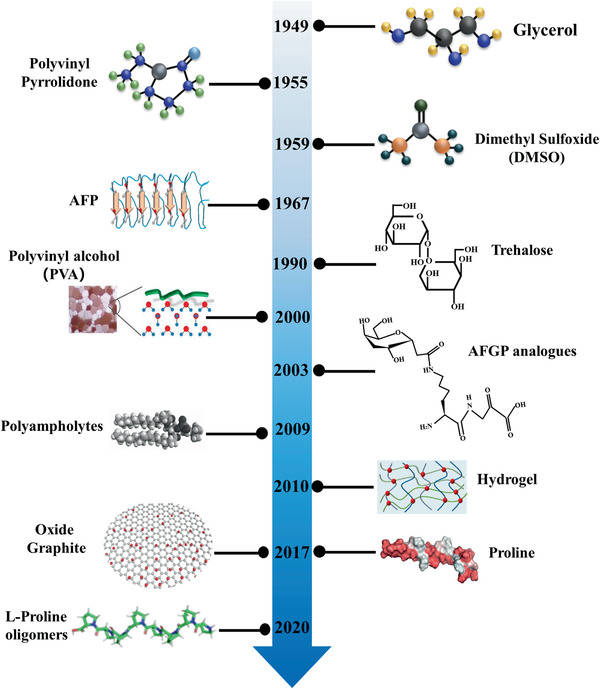
Brief development chronology of chemical ice‐inhibition molecules for cryopreservation. Reproduced with permission.^[^ ^58^ ^]^ Copyright 2013, American Chemical Society. Reproduced with permission.^[^ ^59^ ^]^ Copyright 2016, American Chemical Society. Reproduced with permission.^[^ ^60^ ^]^ Copyright 2017, Wiley‐VCH.Reproduced with permission.^[^ ^113^ ^]^Copyright 2020, American Chemical Society.

Based on the above analysis of ice injury during cryopreservation, it is obvious that effective ice control can weaken the effects of ice injury and improve the efficiency of cryopreservation of cells, tissues, and organs. With the rapid development of materials and chemistry, novel materials with the function of ice tuning have been developed and utilized as ice inhibitors in the process of cryopreservation for a long time (**Figure** **2**), providing great opportunities for the improvement of cryopreservation. Herein, we briefly summarize the ice‐inhibition materials, including CPAs, AFPs, synthetic polymers, nanomaterials, and hydrogels, emphasizing their ice regulation and inhibition mechanisms during cryopreservation.

### 3.1 CPAs

As analyzed in the previous section, the chemical composition of the solution plays a crucial role in the process of freezing and thawing for the successful cryopreservation of various biological samples. Although avoiding ice formation is impossible, strengthening the freezing tolerance of the solution and reducing the ice damage to cryopreserved cells can be achieved.^[^ ^61^ ^]^ To this end, CPAs are used as additives to prevent ice injury in cells. The CPA has received great attention since glycerol was first discovered in 1949.^[^ ^62^ ^]^ Current commercial CPAs are mainly classified into permeating and nonpermeating types, depending on whether they can permeate into cells. As listed in **Table** **1**, the common CPAs have become an indispensable part of the practical application of cryobiology.

**Table 1:** The brief classification and summary of CPAs

Permeating agents	Nonpermeating agents	
Small molecules	Sugars	Polymers
Dimethyl sulphoxide	Sucrose	Polyethylene glicol
Ethylene glycol	Trehalose	Polyvinyl pyrrolidone
Propylene glycol	Raffinose	Hydroxy ethyl starch
Glycerol	Mannitol	Ficoll
Methanol	Glucose	Serum proteins (mixture)
Ethanol	Galactose	Milk proteins (mixture)
Glycine betaine		

**Figure 3:**
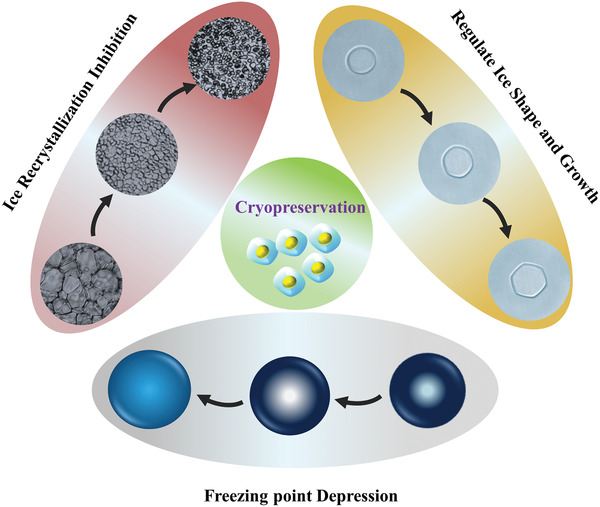
Basic ice‐modulation functions of CPAs. Ice freezing point depression, control of ice shape and growth, and inhibition of ice recrystallization. Reproduced with permission.^[^ ^70^ ^]^ Copyright 2017, Wiley‐VCH.

It is widely believed that CPAs have three functions to achieve ice inhibition and promote cryopreservation: ice‐recrystallization inhibition (IRI), freezing point depression, and ice shape and growth control, as presented in **Figure** **3**. The most common CPA, dimethyl sulfoxide (DMSO), shows the function of ice‐point depression at certain concentrations.^[^ ^63^ ^]^ Meanwhile, the special molecular interaction between DMSO and water alters the freezing process and protects cryopreserved samples. To understand the interaction mechanism of DMSO at the molecular level, Zhang et al. conducted a molecular dynamic simulation between DMSO and water, and the results demonstrated that DMSO could form hydrogen bonds, thus increasing the fraction of unfrozen water and strengthening the freezing tolerance.^[^ ^64^ ^]^ In addition, the number of formed hydrogen bonds gradually increased as the concentration of CPA increased and the temperature decreased, implying that the special interaction limited the diffusion of water molecules in the freezing process. However, the permeating CPAs probably show toxicity, and even have a side effect on the genes of cryopreserved cells at excess concentration, which restricts DMSO's widespread use in clinical applications.^[^ ^65^, ^66^, ^67^ ^]^ Compared with traditional penetrating CPAs, nonreducing sugars, such as nontoxic and biocompatible trehalose and sucrose, possess hydration and are utilized as ice‐inhibition materials.^[^ ^45^ ^]^ Recently, it was discovered that sugar can regulate the shape and delay the growth of ice crystals.^[^ ^68^ ^]^ Additionally, sugars also showed ice‐recrystallization inhibition properties, thus promoting more efficient cryopreservation.^[^ ^69^ ^]^ Currently, in order to solve the contradiction between the toxicity of the permeating CPAs and cryopreserved efficiency, the combination of permeating CPAs and nonpermeating sugar is an effective strategy to decrease the concentration of permeating CPAs and reduce their toxicity, enhancing cryopreserved cells and tissues.

### 3.2 AFPs

**Figure 4:**
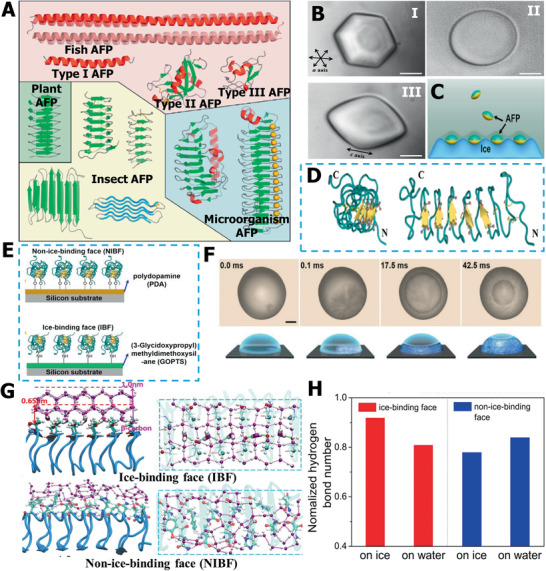
Ice‐regulation functions of AFP. A) The summary of AFPs with different structures. Reproduced with permission.^[^ ^73^ ^]^ Copyright 2014, Elsevier. B) The DIS function of AFPs. C) Probable mechanism of AFPs for ice growth inhibition. B,C) Reproduced with permission.^[^ ^39^ ^]^ Copyright 2016, American Chemical Society. D) The different ice‐binding sites on AFPs structure. E) Different faces of AFPs selectively absorb onto PDA and GOPTS surface. F) The typical ice nucleation images on different AFP faces. G) Molecular dynamic simulation results of different AFP faces with water/ice molecules. H) The comparison of hydrogen bonds of IBFs and BIBFs with water/ice. D–H) Reproduced with permission.^[^ ^85^ ^]^ Copyright 2016, Proceeding of the National Academy of Sciences of the United States of America.

Organisms that live in extremely cold environments possess a special cold‐adaptation ability, producing a type of protein to help them survive.^[^ ^71^ ^]^ The protective proteins, called AFPs, have unique ice‐crystal control functions. The first ice‐binding AFP was reported in the late 1960s and immediately attracted scientific interest, as it can prevent cryoinjury upon exposure to cold temperature conditions.^[^ ^72^ ^]^ To date, various AFPs have been found in the body of natural fish, insects, bacteria, etc.^[^ ^73^ ^]^ As illustrated in **Figure** **4**A, the 3D structure of AFPs is variable and closely associated with their ice‐inhibition activity.^[^ ^74^ ^]^ Generally, AFPs reveal three key macroscopic ice‐tuning properties: dynamic ice shaping (DIS), thermal hysteresis (TH), and IRI.^[^ ^39^ ^]^ The unique ice‐modifying functions of AFPs with respect to DIS and TH are research hotspots and attract significant scientific attention.^[^ ^75^ ^]^ The underlying mechanism of regulating special ice morphologies for AFPs was ascribed to their absorption‐inhibition effect.^[^ ^76^ ^]^ AFPs are capable of binding onto the basal or prism plane of ice crystals and lowering ice growth rates, thus shaping the ice, as shown in Figure 4B. In addition, AFPs can facilitate TH activity, owing to the Kelvin effect.^[^ ^77^ ^]^ The elevated melting point and the depressed freezing point result in temperature hysteresis, which can be explained by the curved and flat ice crystals, according to thermodynamic principles. Interestingly, the hyperactive AFPs possess larger TH gaps, meaning that TH can assess the ice‐tuning capability of AFPs. Ice recrystallization is a process in which smaller ice crystals gradually grow into larger ones at high subzero temperatures. It is a thermodynamically driven process that leads to a decrease in the free energy of the overall system.^[^ ^73^ ^]^ It was proved that the occurrence of ice recrystallization resulted in dehydration and structural and functional damage to the surrounding tissues of organisms. As schematically illustrated in Figure 4C, the AFPs prefer to absorb onto the ice crystal's surface.^[^ ^39^ ^]^ Consequently, the preferential binding between the AFPs plane and ice crystal leads to microcurvatures of the ice surface, thus suppressing the growth of the ice crystals. Molecularly, the hydroxyl groups on AFPs can form hydrogen bonds with ice, thus driving the binding effect of AFPs.^[^ ^78^, ^79^, ^80^, ^81^ ^]^ The solution exhibits decreased ice‐grain area after adding trace amounts of AFPs.

Another topic, on the effects of AFPs on ice nucleation, is interesting, although many research reports about the influence of AFPs on ice nucleation formation remain debatable.^[^ ^82^, ^83^, ^84^ ^]^ AFPs possess two faces: an ice‐binding face (IBF) and a nonice‐bind‐face (NIBF), as revealed in Figure 4D. Recently, Wang and co‐workers elucidated the Janus effect of AFPs on ice nucleation, which gave different ice nucleation tuning effects of IBF and NIBF at the molecular level.^[^ ^85^ ^]^ First, the hyperactive AFP obtained from a beetle was selectively absorbed on a solid substrate. Briefly, the IBF was absorbed by polydopamine and the NIBF was exposed. Furthermore, the NIBF can be bound by (3‐glycidoxypropyl) methyldimethoxysilane (GOPTS), as shown in Figure 4E. Different face effects of AFPs on the nucleation time and temperature of the droplets were assessed (Figure 4F). The results revealed that ice nucleation was facilitated once the IBF was exposed to liquid water, but the ice nucleation would be restricted if the NIBF came into contact with liquid water. As illustrated in Figure 4G, the molecular dynamic simulation further demonstrated that IBFs can exhibit ordered hexagonal ice‐like water due to the occurrence of hydrophobic methyl groups and hydrophilic groups; there was no ordered water structure when NIBFs were exposed to liquid water, because of the irregularly arranged functional groups and the presence of charged groups. Meanwhile, the quantitative assessment of hydrogen bonds proved that liquid water was more stable in NIBFs, while the energy of ice crystals was more stable in IBFs (Figure 4H). Hence, it can be concluded that ice nucleation is determined by ice‐binding sites, which provides deep insights into the AFPs’ modulation ability on ice‐crystal nucleation.

Based on the above‐mentioned unique ice‐control properties, AFPs have become a promising ice‐inhibition candidate for use in cryopreservation.^[^ ^86^ ^]^ The first application of AFPs for cryopreservation began in 1990; Rubinsky et al. discovered that the addition of AFPs could increase the cold‐tolerance of porcine oocytes at hypothermic temperature.^[^ ^87^ ^]^ Since then, various AFPs have been used in cryopreservation. In another experiment, Lee et al. attempted to apply type III AFPs in the vitrification cryopreservation of mouse ovaries, and the results revealed that the ovaries treated with AFPs possessed higher intact‐follicle ratios than in the control group, implying the positive effect of AFPs in vitrification cryopreservation.^[^ ^88^, ^89^ ^]^ Until now, the main cryoprotective mechanism of AFPs in cryopreservation is ascribed to their IRI ability and interaction with cell membranes. We can conclude that AFPs reduce the extracellular ice damage and protect the integrity of cell membranes in freeze–thaw cycles.^[^ ^90^ ^]^ More recently, to explore the intracellular role of AFPs, Gibson and co‐workers delivered type III AFP into the intracellular location.^[^ ^91^ ^]^ They found that the excess AFP can cause toxic effects to cryopreserved samples and even lead to gene variation, thereby limiting their widespread applications in the field of cryopreservation.^[^ ^92^, ^93^ ^]^ With the rapid development of structural biology, a deep understanding of the correlation between the 3D structure and functions of AFPs will elucidate their ice‐regulation mechanism and facilitate their practical application in the field of cryopreservation.

### 3.3 Synthetic Polymer

While AFPs show unique modulation and modifying functions for ice crystals, they are not always appropriate for cryopreservation, owing to drawbacks such as high cost, potential immunogenicity and toxicity effects, large‐scale production, and needle‐shaped ice crystals.^[^ ^40^ ^]^ In particular, for needle ice, the curvature of ice crystals cannot be influenced without antifreeze glycoproteins (AFGPs), and finally, the shape of round flat ice crystals is generated. Once the AFGPs absorb onto the surface of a particular plane of ice, the special surface curvature will increase, resulting in vapor pressure, a subsequent decrease in the melting point, and water molecules being unable to assimilate into surface pockets on the ice.^[^ ^40^, ^73^ ^]^ The AFGPs absorption onto the fast‐growing prism facilitates ice growth from the basal plane direction of the ice, producing needle‐shaped ice crystals. It is self‐evident that the needle‐shaped ice crystals can lead to serious mechanical injury for cryopreserved samples when used during cryopreservation. Therefore, it is crucial to develop an artificial synthetic polymer with an ice‐tuning function to overcome the drawbacks of the AFGPs. Currently, with the rapid developments in polymer chemistry, significant progress has been made in designing and synthesizing complex architectures and functional groups, providing a broad platform for achieving AFP‐like polymers but with scalable and stable ice‐modulation functions. Recently, variable small molecular and macromolecular polymers have been introduced into the emerging field of ice inhibition, and thus, have great potential for cellular cryopreservation. Herein, we aim to focus on synthetic anti‐icing polymer materials, including small molecules, ice binding, and amphiphilicity polymers.

**Figure 5:**
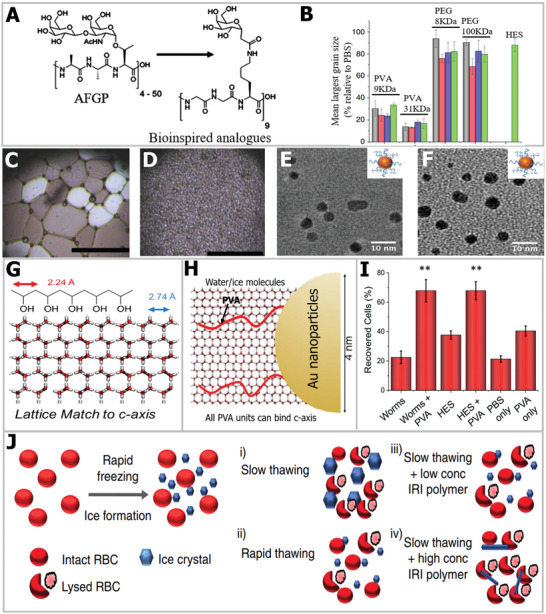
Ice‐inhibition performances of AFGP analogs and PVA. A) Structure simplification to AFGP analogs with IRI ability. Reproduced with permission.^[^ ^95^ ^]^ Copyright 2003, Springer Nature. B) IRI comparison of PVA with other polymers used for cryopreservation. C) Cryomicroscopic image of ice crystal without PVA. D) Cryo‐microscopic image of ice crystals with PVA. B–D) Reproduced with permission.^[^ ^102^ ^]^ Copyright 2014, Springer Nature. E) TEM image of PVA_140_–Au. F) TEM image of PVA_98_–Au. G) Lattice match of PVA with prime plane of water. H) Au does not disturb the pattern match of PVA with water. E–H) Reproduced with permission.^[^ ^107^ ^]^ Copyright 2018, American Chemical Society. I) The influence of PVA on cell recovery post‐thaw. J) Proposed mechanism of PVA for IRI and enhancing cell cryopreservation. Reproduced with permission.^[^ ^102^ ^]^ Copyright 2014, Springer Nature.

Based on hydration, a series of small molecular carbohydrates have been synthesized.^[^ ^94^ ^]^ Ben and co‐workers first reported different small‐molecule AFGP analogs as ice inhibitors in 2003;^[^ ^95^ ^]^ the correlation between carbohydrate hydration and IRI was assessed. They found that the active carbohydrate moiety on C‐linked antifreeze glycoprotein analogs contributed to their IRI ability (**Figure** **5**A). The underlying IRI mechanism was analyzed and can be explained as follows: in the process of ice formation, a semiordered quasi‐liquid layer existed between the ice lattice and bulk water.^[^ ^96^, ^97^ ^]^ Then, the bulk water transformed into a quasi‐liquid layer, finally leading to IRI. Carbohydrates can disturb 3D hydrogen bonds in bulk water, thus increasing the energy of bulk water transfer into the quasi‐liquid layer, consequently resulting in the IRI. More recently, Ben and co‐workers designed a new class of small molecules, *N*‐aryl‐d‐aldonamides.^[^ ^98^ ^]^ Based on ice growth control and IRI, the small‐molecule additives facilitated the cryopreservation efficiency of hematopoietic progenitors. Besides, Ben's laboratory reported various other carbohydrate derivatives, peptides, and glycopeptides as ice inhibitors, which have been summarized in previous reviews.^[^ ^40^, ^99^, ^100^ ^]^

In addition to small molecules, synthetic macromolecule polymers can be assembled from vast monomers, thereby endowing the polymers with flexible structures and functions. At present, as a typical ice inhibitor, polyvinyl alcohol (PVA) exhibits great potential for ice growth inhibition, and has been utilized for IRI despite its molecular structure differing from that of native AFP. Knight et al. first discovered that the IRI property of PVA that did not show TH performance.^[^ ^101^ ^]^ Since that time, PVA has been widely studied in the field of ice inhibition. As shown in Figure 5B, compared with other commercial polymers, such as PEG and hydroxyethyl starch (HES), applied in cryopreservation, PVA showed a more significant IRI effect.^[^ ^99^ ^]^ In particular, the IRI activity of PVA is greatly dependent on its polymerization.^[^ ^58^ ^]^ More recently, Naullage and Molinero used molecular and numerical simulations and found that the IRI activity of PVA occurred when the degree of PVA polymerization was between 15 and 20.^[^ ^103^ ^]^ This also indicates that a lower degree of polymerization contributes to the high‐IRI activity of PVA. Additionally, the molecular dynamic simulation results indicated that the tacticity of hydroxyl groups was a crucial factor in determining the IRI activity of PVA.^[^ ^104^, ^105^ ^]^ For instance, by changing the structure of PVA backbones, the experimental results showed that the atactic PVA exhibited more significant IRI ability compared with isotactic PVA, indicating that the tacticity of hydroxyl groups is an essential factor for the IRI activity of PVA. The molecular dynamic simulation results showed that the atactic PVA can spread better at the ice–water interface, and possessed a stronger affinity to the ice and water surface compared with isotactic PVA, resulting in a larger covered area of ice crystals for atactic PVA and leading to more effective IRI capability. With respect to the IRI mechanism, Budke and Koop first proposed an adsorption model for PVA.^[^ ^106^ ^]^ The absorption mechanism can be described as follows: the arrangement of water molecules can be recognized by PVA molecules, and the PVA molecules can further adsorb onto the primary and secondary prism faces of hexagonal ice through hydrogen bonds. Additionally, Gibson's group has also put great effort into the IRI ability of PVA and its application in cryopreservation.^[^ ^100^ ^]^ Figure 5C,D exhibits the IRI ability of PVA, and it can be seen that the ice‐grain area of the PBS solution sharply decreased after its addition.^[^ ^99^ ^]^ Interestingly, they further immobilized the two kinds of PVA (PVA_140_ and PVA_98_ mean *M_n_* = 12 000 and 3900 g mol^−1^) onto a gold nanoparticle surface, as shown in Figure 5E,F, and the PVA–Au composite still retained IRI activity, which opens a new avenue for developing multifunctional IRI materials.^[^ ^107^ ^]^ Generally, the atomic distance between the OH groups of PVA is essential for the structure to match with water and ice molecules. In this respect, Gibson and co‐workers showed that the distance between hydroxyls on PVA (2.92 Å) can precisely match with the prismatic plane of the ice crystals (2.74 Å), as illustrated in Figure 5H, which probably endowed the PVA with macroscopic IRI activity. For the PVA granted on Au, the low granted density had no influence on the IRI ability of PVA (Figure 5G). On the basis of PVA's IRI effect, they further combined PVA with copolymer and achieved enhanced IRI effect, increasing the cryopreservation efficiency (Figure 5I), thus first realizing solvent‐free cryopreservation for red blood cells.^[^ ^108^ ^]^ Briefly, PVA can suppress the extracellular ice and reduce the ice‐recrystallization damage to cells in the thaw process, as illustrated in Figure 5J.^[^ ^102^ ^]^ In addition to red blood cells, they also utilized PVA against ice injury and realized glycerol‐free cryopreservation for proteins.^[^ ^109^ ^]^ Furthermore, it has been reported that PVA can inhibit ice nucleation and facilitate vitrification cryopreservation.^[^ ^110^ ^]^ In summary, as a well‐recognized ice inhibitor, the IRI of PVA polymer and its further optimization will have positive effects on the cryopreservation field.

**Figure 6:**
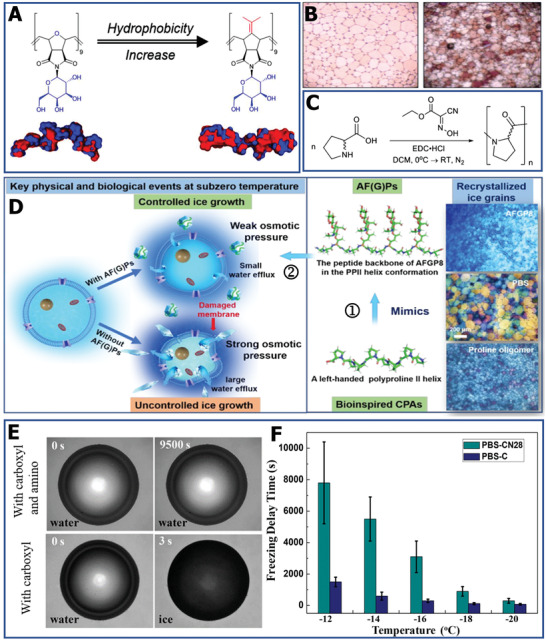
Ice‐modulation properties of different polymers. A) Increase in the hydrophobic surface map of copolymer. Reproduced with permission.^[^ ^112^ ^]^ Copyright 2018, American Chemical Society. B) The IR cryomicrograph of PBS solution without (left) and with polyproline (right). C) The polymerization and molecule structure of polyproline. B,C) Reproduced with permission.^[^ ^58^ ^]^ Copyright 2017, Wiley‐VCH. D) The schematic diagram of the IRI mechanism of polyproline for cryopreservation. Reproduced with permission.^[^ ^113^ ^]^ Copyright 2020, American Chemical Society. E) The image of ice nucleation of polyampholytes with different functional groups. F) Comparison of ice nucleation delay time for different polyampholytes. E,F) Reproduced with permission.^[^ ^116^ ^]^ Copyright 2017, American Chemical Society.

Mimicking the 3D structure of an AFGP offers another strategy to attain ice inhibitors. For native AFP or AFGP, it has been shown that the separated hydrophobic/hydrophilic groups are also a crucial factor for effective ice inhibition. Precious modulation and design of the hydrophobic/hydrophilic domains are essential. Recent research has made remarkable progress in molecular modeling and experiments to elucidate the structure–property relationship of synthetic polymers with ice‐modifying functions.^[^ ^111^ ^]^ For example, Graham et al. designed a type of facial glycopolymer that contained separate hydrophobic and hydrophilic faces.^[^ ^112^ ^]^ In order to increase the hydrophobicity, some comonomers were incorporated into the polymer, increasing their solubility and IRI activity (**Figure** **6**A). By mimicking the amphipathic helix structure of AFGP,^[^ ^112^ ^]^ they found the IRI ability of polyproline (Figure 6B). As demonstrated in Figure 6C, there is no amide N–H in polyproline, which means that it cannot form intramolecular bonds.^[^ ^60^ ^]^ However, on the one hand, the separated hydrophilic and hydrophobic domains of amphipathic polyproline possess a PP II helix structure and imitate the AFGP, which is associated with its ice‐inhibition activity.^[^ ^60^, ^100^ ^]^ On the other hand, the ice‐inhibition mechanism of facially amphipathic materials is also dependent on the local water ordering, rather than ice binding, unit.^[^ ^109^ ^]^ In addition to the structural similarity, the charge anisotropy of the amphipathic molecular structure is also an important factor for suppressing ice growth.^[^ ^111^ ^]^ Based on the IRI activity, the polyproline further promoted cryopreservation recovery, demonstrating a new macromolecular additive for realizing high‐efficiency storage. Most recently, Wang and co‐workers applied the l‐proline oligomer (50 × 10^−3^ m l‐Pro_8_, a monomer concentration of 0.4 m) for more successful cryopreservation of oocytes (99.11%) and reduced the CPA concentration from 4.3 to 2.5 m.^[^ ^113^ ^]^ More importantly, the mitochondrial function of oocytes postcryopreservation was also improved, indicating that the addition of l‐Pro_8_ can decrease the extracellular ice injury during the freeze–thaw process. The proposed mechanism was attributed to the unique ice growth control of proline, as illustrated in Figure 6D. Notably, the ice‐inhibition mechanism of facially amphipathic materials should be ascribed to their interaction with the quasi‐liquid layer or bulk water rather than ice binding.

Moreover, Matsumura and co‐workers observed that polyampholytes are also effective ice inhibitors, and enable solvent‐free cryopreservation of stem cells.^[^ ^59^, ^114^, ^115^ ^]^ Based on this effect, the influence of polyampholytes on IRI was studied. The underlying mechanism may be that the charge‐balanced nature feature of polyampholytes probably disturbs the quasi‐like water between the water and ice interface, inhibiting the growth of ice crystals. In addition to IRI, it has been found that polyampholytes can be used to inhibit ice nucleation. By regulating the structure of polyampholytes, carboxylated polyampholytes and polyampholytes with carboxyl and amino groups were synthesized. As shown in Figure 6E, there was no visible ice formation until water condensed continually in the 9500 s for the polyampholytes with amino groups.^[^ ^116^ ^]^ At the same time, polyampholytes with carboxyl and amino groups can delay the freezing time from 400 ± 150 s to 7800 ± 2600 s when the temperature decreases from −12 to −20 °C (Figure 6F). By delaying ice nucleation, polyampholytes can reduce the devitrification injury for cryopreserved cells during the thawing process, which is necessary for cell survival. Overall, a range of synthetic polymers with ice‐modulation functions has been developed, but it should be noted that the state‐of‐the‐art IRI agents should also have excellent biodegradability and biocompatibility for further practical cryopreservation application.^[^ ^100^, ^117^, ^118^, ^119^, ^120^, ^121^ ^]^

### 3.4 Nanomaterials

**Figure 7:**
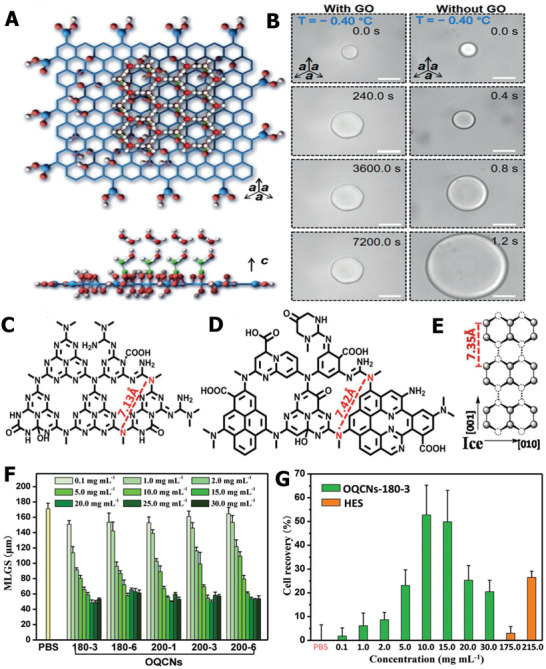
Ice‐inhibition performance of GO and OQCNs and their applications for cell cryopreservation. A) The honeycomb‐like structure of GO matched with ice lattice and the formation of hydrogen bonds between ice crystals and hydroxy groups. B) The influence of GO on the growth and shape of ice crystals. A,B) Reproduced with permission.^[^ ^123^ ^]^ Copyright 2016, Wiley‐VCH. C) Molecule structure of OCNs. D) Molecule structure of OQCNs. E) Primary prism face of hexagonal ice crystal. F) Comparison of the ice‐grain area with different concentrations of OQCN in PBS solution. G) The quantitative assessment of cryopreservation efficiency using OQCNs. C–G) Reproduced with permission.^[^ ^70^ ^]^ Copyright 2017, Wiley‐VCH.

Similar to the functions of AFP and AFGP, some nanomaterials also possess ice‐regulation properties and were added as ice‐inhibition agents in the process of cryopreservation. One typical example is graphene oxide (GO), which shows a similar structure to that of natural AFP; that is, the distance between each monomer is almost the same as that of the GO lattice, which offered a suitable reason for the ice‐inhibition capability of GO.^[^ ^122^ ^]^ Geng et al. first discovered the ice‐tuning abilities of GO, and elucidated its intrinsic mechanism through experiments and molecular dynamic simulation strategy.^[^ ^123^ ^]^ The experimental results demonstrated that GO can control ice shape and growth, and has great potential for application in cryopreservation. The honeycomb‐like structure of GO can match with the ice lattice and the hydrogen bonds formed between the water/ice molecules and hydroxyl groups on the top surface of GO, as schematically illustrated in **Figure** **7**A. In contrast to pure water, GO significantly suppressed the growth and regulated the shape of single ice crystals. Hexagonal ice was found in the GO dispersion, whereas a disk‐shaped ice crystal was observed in pure liquid water, as shown in Figure 7B. It is worth noting that the degree of oxidation and the size of GO had a profound effect on the ice‐control ability. Recently, it was reported that different sizes of GO are associated with the nucleation temperature of ice.^[^ ^122^ ^]^ For the same size of GO, the increase in carboxyl groups can result in a more obvious ice‐inhibition effect, which indicates that the carboxyl groups play a crucial role in tuning ice. Similar to the absorb‐inhibition mechanism of AFP, GO can also bind to the basal or prism plane of ice crystals. The results of the molecular dynamics simulation also showed that more hydrogen bonds formed between GCOOH and ice‐like water than liquid water. Based on the ice‐regulation capability of GO, it was discovered that GO exhibited IRI both in NaCl solution and culture medium; thus, it was used as an additive in cryopreservation to reduce ice injury. In stark contrast, the cryopreservation efficiency of horse sperm can increase from 24.3% to 71.3% after adding only 0.01 wt% GO.

In addition to GO, different types of oxide quasi‐carbon nitride quantum dots (OQCNs) also showed TH, ice shaping, and IRI effects.^[^ ^70^ ^]^ The hydrogen bond formation between OQCNs and ice is significant for the ice‐control functions. Interestingly, the OCNs are not capable of exhibiting ice regulation, which can be explained by the difference in atomic arrangement between OCNs and OQCNs, as shown in Figure 7C,D. Molecularly, the OQCNs atomic distance between O and N atoms (7.42 Å) matched better than that of OCNs (7.13 Å) with an ice‐crystal lattice (Figure 7E), thus leading to a more obvious ice‐suppression ability. In addition, the OQCNs can prefer to bind to the surface of ice crystals and cause microcurvatures to ice, possessing a similar Kelvin effect to that of AFP. On the basis of the excellent ice‐inhibition function for PBS solution (Figure 7F), the OQCNs were used as CPAs in the cryopreservation of red blood cells. The results revealed that the viability of red blood cells increased up to 55% without any other commercial CPAs, which is more efficient than the results after the addition of traditional HES (≈25%), as illustrated in Figure 7G. Due to the extracellular ice‐inhibition activity of OQCNs and GO, the cryopreserved cells underwent less ice damage in the freeze–thaw cycles, leading to the improvement of survival viability. With the rapid development of nanomaterials, other carbon‐based nanoparticles such as modified graphene, oxide carbon nanotubes, and oxidized quasi‐carbon nitride quantum dots have been prepared to explore their influence on ice nucleation or growth.^[^ ^124^, ^125^, ^126^, ^127^ ^]^

**Figure 8:**
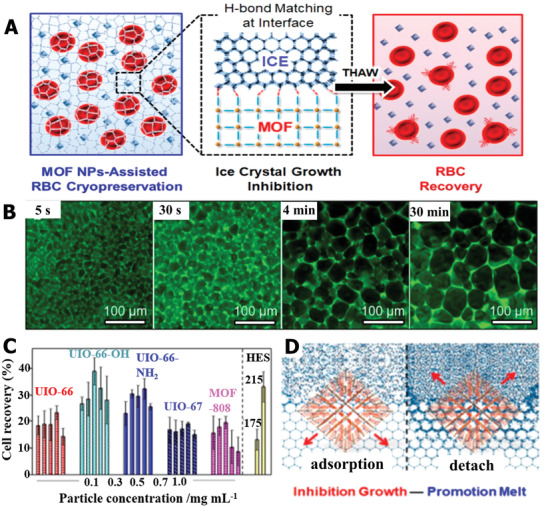
The ice‐suppression function of MOF nanoparticles facilitated red blood cell cryopreservation. A) Schematic illustration of MOF nanoparticles improving cryopreservation efficiency of red blood cells based on ice inhibition. B) Images of ice‐crystal growth at different times for the PBS solution with MOF nanoparticles. C) The cell recovery comparison in HES and MOF with different functional groups and concentrations. D) The possible mechanism of ice inhibition and melt enhancement for MOF. A–D) Reproduced with permission.^[^ ^128^ ^]^ Copyright 2019, American Chemical Society.

Hydrogen bonding interactions play an important role in ice modulation. By designing the surface functional groups to regulate the surface chemistry specificity of nanomaterials, it is feasible to achieve a precise hydrogen bonding taht can match with ice‐crystal planes. In a more recent study, zirconium (Zr)‐based metal–organic framework (MOF) nanoparticles were used to suppress ice recrystallization and melt ice crystals.^[^ ^128^ ^]^ As illustrated in **Figure** **8**A, the periodic arrangement of organic linkers on the MOF can offer hydrogen donors to form hydrogen bonds at the ice interface, and the MOF nanoparticles can become effective additives to promote the cryopreservation of red blood cells. In the recrystallization process, the ice‐grain size of the PBS solution with MOF nanoparticles increased rapidly in the initial stage, and after 15 min, the increase in crystallization almost stopped (Figure 8B). Compared with pure PBS solution without MOF nanoparticles, the ice size continuously grew for 25 min and, consequently, showed a much larger ice area. Based on this ice‐inhibition performance of MOF nanoparticles, the MOF can show higher cryopreservation efficiency than the commercial polymer HES, as depicted in Figure 8C. Owing to the interaction between the MOF and ice interface, the detachment of MOF nanoparticles was able to release fixed water to a free state, which indicated that the MOF can be a catalyst to accelerate ice melting (Figure 8D). Hence, the flourishing MOF nanomaterials with ice‐regulation functions both in ice inhibition and melting show great potential for cell cryopreservation. To further achieve the practical application of various nanoparticles in clinical cryopreservation, the added nanomaterials should have outstanding biocompatibility and cannot cause any toxicity to cryopreserved biological samples. Briefly, the innovation of chemical synthesis can endow more nanomaterials with active ice‐inhibition ability and facilitate the development of nanomaterials for the application of cryopreservation.

### 3.5 Hydrogel

Recently, a range of hydrogel materials with ice modulation and inhibition functions have been widely researched and utilized for cryopreservation. Hydrogels are commonly semisolid phases with a network structure.^[^ ^129^, ^130^ ^]^ In terms of the interactions between monomers that compose the networks, they can be divided into chemical crosslinking and physical crosslinking methods.^[^ ^131^ ^]^ Chemical crosslinking is mainly based on covalent bonds, and physical crosslinking relies on weaker physical forces such as hydrogen bonds, *π*‐stacking interactions, and van der Waals interactions.^[^ ^132^ ^]^ The distinct network structure and composition endow the hydrogel with outstanding biocompatibility, thermal reversibility, optical clarity, and tunable mechanical properties.^[^ ^133^, ^134^, ^135^ ^]^ Recently, anti‐icing hydrogels have been widely reported. The state of the hydrogel and its chemical composition play an important role in the ice‐inhibition performance of hydrogels. In general, the water in a hydrogel can be classified into three states: free water, weakly bound water (also known as intermediate water), and strongly bound water.^[^ ^136^ ^]^ The strong bound water can maintain its fluidity and remain in a liquid state even when the temperature is below −100 °C, which provides guidance for the design principles of the chemical composition of the hydrogel with ice‐inhibition ability. By introducing ions and alcohol into the hydrogel and modifying its network, the hydrogel can be endowed with ice‐inhibition properties. The underlying mechanisms of hydrogels are mainly attributed to strong interactions such as hydrogen bonds between water molecules and the hydrogel network, thus reducing the fraction of free water.^[^ ^137^ ^]^ Based on this, a hydrogel material with an ice‐suppression function due to its special chemical composition and state has been widely investigated and applied to cell cryopreservation.^[^ ^138^ ^]^

**Figure 9:**
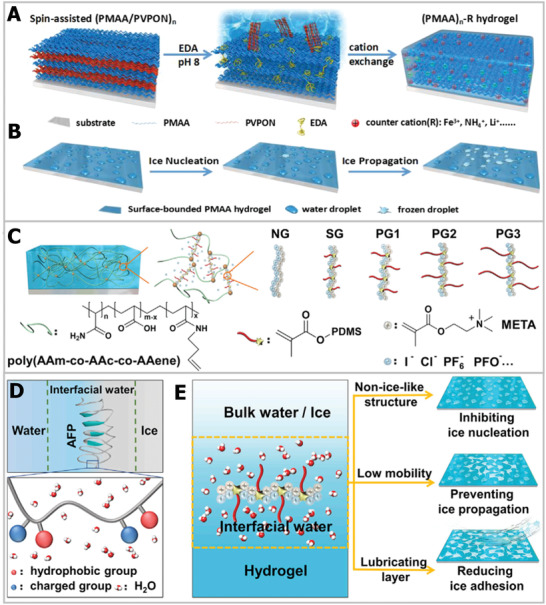
Ice‐inhibition functions based on hydrogel materials. A) The schematic diagram of the hydrogel fabrication process. B) The modulation functions of hydrogel on ice nucleation and propagation. A,B) Reproduced with permission.^[^ ^139^ ^]^ Copyright 2018, American Chemical Society. C) The crosslinked structure of hydrogel. D) Illustration of hydrophobic and hydrogel bonds of AFP‐regulated ice. E) Bioinspired AFP hydrogel tuned ice nucleation, ice propagation, and reduced ice adhesion. C–E) Reproduced with permission.^[^ ^140^ ^]^ Copyright 2020, Elsevier.

Based on the ionic specificity for ice nucleation, Guo et al. designed counterion multilayer hydrogels that showed an inhibitory effect on both ice nucleation and propagation.^[^ ^139^ ^]^ As schematically illustrated in **Figure** **9**A, the hydrogel was first prepared via a hydrogen‐bonded multilayer of polymethacrylic acid (PMAA)/poly(*N*‐vinylpyrrolidone) (PVPON) based on the layer‐by‐layer (LBL) approach. Then, the PMAA layer was crosslinked with ethylenediamine (EDA) by sacrificial template strategy. Based on the counterion effects, the PMAA hydrogel displayed an 11 °C ice nucleation temperature window and delayed four orders of magnitude of ice‐crystal propagation time, as depicted in Figure 9B. The special ice tuning via ionic specialty was attributed to the change in ice‐like water and the formation of hydrogen bonds. He et al. further combined the counterion effect with hydrophobic bionic AFP materials,^[^ ^140^ ^]^ designing hydrophobic polydimethylsiloxane (PDMS) chains with a polyelectrolyte hydrogel (Figure 9C). Bioinspired by the concept of AFP, the hydrophobic functional group can adsorb onto the ice surface (Figure 9D); meantime, the charged group can modulate interfacial water, resulting in the designed hydrogel integrated multifunctional anti‐icing performance, including inhibiting ice nucleation, preventing ice propagation, and reducing ice adhesion (Figure 9E). In general, the optimization and design of composition and molecular structure are crucial factors for enhancing anti‐icing hydrogel materials. For the application potential of the anti‐icing hydrogel in the field of cell cryopreservation, the ice nucleation inhibition ability of the hydrogel can delay ice nucleation formation for the vitrification cryopreservation method, which is of great importance to reduce devitrification cryodamage to cells during the freeze–thaw process. In addition, cell–hydrogel constructs based on cell encapsulation technology, preventing ice propagation, and reducing ice adhesion at the hydrogel interface are critical to restrict the growth and propagation of ice crystals into cells, thereby decreasing ice injury during the cryopreservation process.^[^ ^42^ ^]^

**Figure 10:**
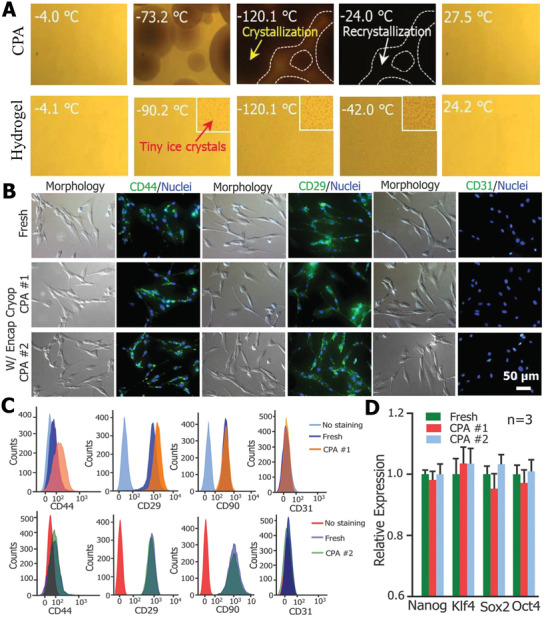
The applications of hydrogel in cell cryopreservation, based on its ice‐inhibition performance. A) Comparison of IRI between alginate hydrogel and CPA in the warming process. Reproduced with permission.^[^ ^146^ ^]^ Copyright 2018, Royal Society of Chemistry. B) Immunostaining of CD44 (+), CD29 (+), and CD31 (−) expression on the recovered cells. C) Flow cytometry assessment of the expression of CD44 (+), CD29 (+), and CD31 (−) expression on the post‐thaw cells. D) Quantitative RT‐PCR analysis of the expression of four stem cell genes in the cells (*n* = 3). B–D) Reproduced with permission.^[^ ^147^ ^]^ Copyright 2017, Wiley‐VCH.

Unlike other ice‐inhibition materials, the hydrogel can offer an excellent physiological environment for cells, and can be regarded as a cell reservoir owing to its sufficient amount of liquid and bionic crosslinked network structure.^[^ ^141^ ^]^ Taking advantage of the anti‐icing and biocompatibility performance, the hydrogel materials show great potential for improving cryopreservation efficiency.^[^ ^142^, ^143^, ^144^ ^]^ For instance, it was observed that alginate hydrogel can improve the survival rate of human embryonic stem cells during slow freezing. Moreover, the ion‐crosslinked alginate hydrogel can also facilitate vitrification, thereby reducing the concentrations of CPA up to 1.5 m, which is attributed to the preferential vitrification of the solution in the alginate hydrogel compared with the bulk solution outside the cells.^[^ ^145^ ^]^ To further explore the ice‐tuning properties of alginate hydrogels in cooling–thawing cycles, Zhang et al. further analyzed the ice‐formation process at different cooling and annealing temperatures.^[^ ^146^ ^]^ They discovered that the alginate hydrogel was able to decrease the ice‐formation temperature and delay ice formation. As shown in **Figure** **10**A, compared with pure CPA, the hydrogel showed smaller ice crystals. Based on the excellent ice‐control permeances, the application of alginate hydrogel in stem cell cryopreservation and functional assessment of recovered stem cells was carried out.^[^ ^147^ ^]^ The viabilities of postencapsulation using alginate hydrogel (>70%) were remarkably higher than without hydrogel (≈20%). Meanwhile, the functional evaluation of recovered stem cells, including stemness and multilineage differentiation, was carried out. The results indicated that the alginate hydrogel used for cryopreservation had no influence on stem cells compared with a fresh group, as exhibited in Figure 10B–D. The protective mechanism of alginate hydrogel toward stem cells can be described as follows. The alginate hydrogel can facilitate the vitrification in the microcapsule and avoid cryoinjury to cells during the cooling process. Meanwhile, the alginate hydrogel inhibits the devitrification and protects cells from ice damage outside the hydrogel during the warming process. In particular, the removal of hydrogel is easy to implement and does not cause any injury to cryopreserved cells. Hence, it is reasonable to believe that the biocompatible and anti‐icing hydrogel will play an increasingly important role in the cryopreservation field.

## 4 Engineering Strategies for Ice Inhibition

Various flourishing engineering strategies offer new paths to inhibit ice and facilitate the efficiency of cryopreservation. In the next section, the promoted cryopreservation based on state‐of‐art ice‐inhibition strategies will be analyzed from the perspective of trehalose delivery, cell encapsulation, and bioinspired structure design.

### 4.1 Trehalose Delivery for Intracellular Ice Inhibition

Intracellular ice formation is a major factor for cell death in the process of cryopreservation.^[^ ^47^ ^]^ Currently, the common permeating CPAs such as DMSO can inhibit cryoinjury by hydration and modulate ice formation and growth. However, DMSO is toxic to cells and can cause protein degeneration and gene variation. Moreover, permeating CPAs are difficult to remove from cells, indicating the significance of using nontoxic and new‐type ice‐inhibition materials for weakening, and even avoiding, intracellular ice injury.^[^ ^34^, ^99^ ^]^ Trehalose, a biocompatible sugar present in nature, has been utilized as an alternative CPA.^[^ ^45^, ^148^ ^]^ Trehalose can protect cells from cryoinjury two ways: 1) enhancing hydration and forming hydrogen bonds, inhibiting ice formation, and 2) forming a glass matrix to restrict metabolic activity.^[^ ^69^, ^149^, ^150^, ^151^, ^152^ ^]^ However, trehalose cannot penetrate cell membranes and, thus, acts as an extracellular ice‐inhibition agent.^[^ ^153^ ^]^ Recently, a series of strategies have been explored to deliver trehalose into cells to reduce intracellular ice damage and improve cryopreservation efficiency.^211^ Generally, delivery methods can be classified into physical and chemical approaches.^[^ ^154^ ^]^ These trehalose‐delivery methods have been reviewed in previous studies.^[^ ^34^, ^149^, ^154^ ^]^ In the next section, we will focus on the novel nanoparticle‐mediated trehalose delivery strategy, and provide several of the latest examples.

**Figure 11:**
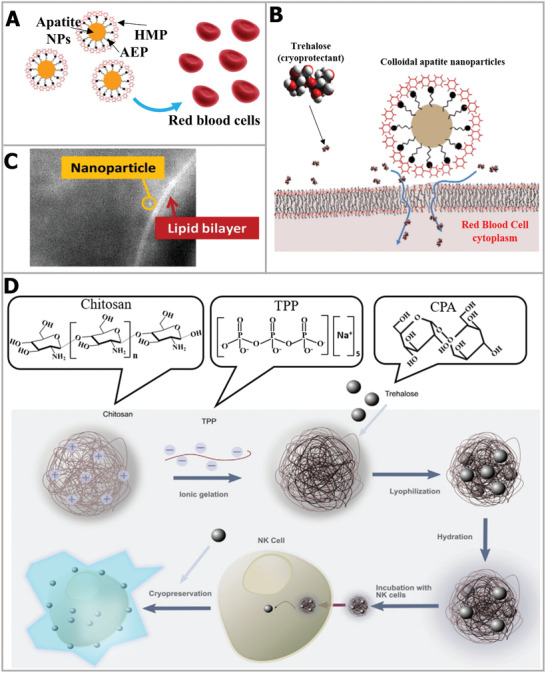
Trehalose‐delivery strategy based on interaction with cell membrane. A) Schematic illustration of nanoparticles. B) The mechanism of nanoparticles inducing trehalose delivery by interacting with the cell membrane. C) Epifluorescence image of fluorescing Eu‐doped apatite NP next to lipid bilayer. A–C) Reproduced with permission.^[^ ^158^ ^]^ Copyright 2017, Elsevier. D) The nanoparticles assembly process and delivery to cells. Reproduced with permission.^[^ ^159^ ^]^ Copyright 2020, Wiley‐VCH.

Nanomaterials have been used as vehicles for the delivery of drugs in the medical field, especially for the release of small molecules for targeted therapy of cancer cells.^[^ ^155^, ^156^, ^157^ ^]^ Hence, the previous research offers a referential strategy for the delivery of trehalose into cells. In brief, the nanoparticle‐mediated method can induce interactions with the cell membrane and promote membrane permeability. For example, Stefanic et al. designed colloidal bioinspired apatite nanoparticles and promoted trehalose delivery into red blood cells.^[^ ^158^ ^]^ As shown in **Figure** **11**A, trehalose was encapsulated by aminoethylphosphate (AEP) and hexametaphosphate (HMP). The formation of the cover layer was attributed to the stability between the HMP anions and AEP cations. In contrast to other mammalian cells, endocytosis does not exist in red blood cells. The possible mechanism of trehalose delivery assisted by nanoparticles was the change in physical performance due to the local interaction between the cell membrane and apatite nanoparticles, as illustrated in Figure 11B. It was noted that the apatite nanoparticles did not permeate the cell membrane, which can be proved by the image of fluorescently labeled nanoparticles (Figure 11C). The cryopreservation efficiency of red blood cells using trehalose delivery was up to 92%, which was 42% higher than that without apatite nanoparticle assistance, and comparable to the survival rate using toxic glycerol as CPA. More recently, in order to avoid intracellular ice damage, Matosevic and co‐workers achieved high‐efficiency DMSO‐free cryopreservation of natural killer (NK) cells by using nanoparticle‐mediated intracellular trehalose delivery.^[^ ^159^ ^]^ As schematically illustrated in Figure 11D, chitosan–tripolyphosphate (CS–TPP) nanoparticles were assembled by ionic gelation. In the acidic environment of the cell interior, the CS–TPP nanoparticles can successfully release trehalose, owing to the pH‐responsive property. By inhibiting intracellular ice formation, the viability of NK cells using CS–TPP nanoparticles and DMSO cryopreservation were comparable. More importantly, trehalose‐delivered NK cells still maintained normal function, including effective cytotoxic, degranulation, and cytokine production functions against tumor targets. There is no doubt that the NK cell cryopreservation based on nanoparticle‐mediated intracellular trehalose delivery will promote cell‐based immunotherapies.^[^ ^160^ ^]^

**Figure 12:**
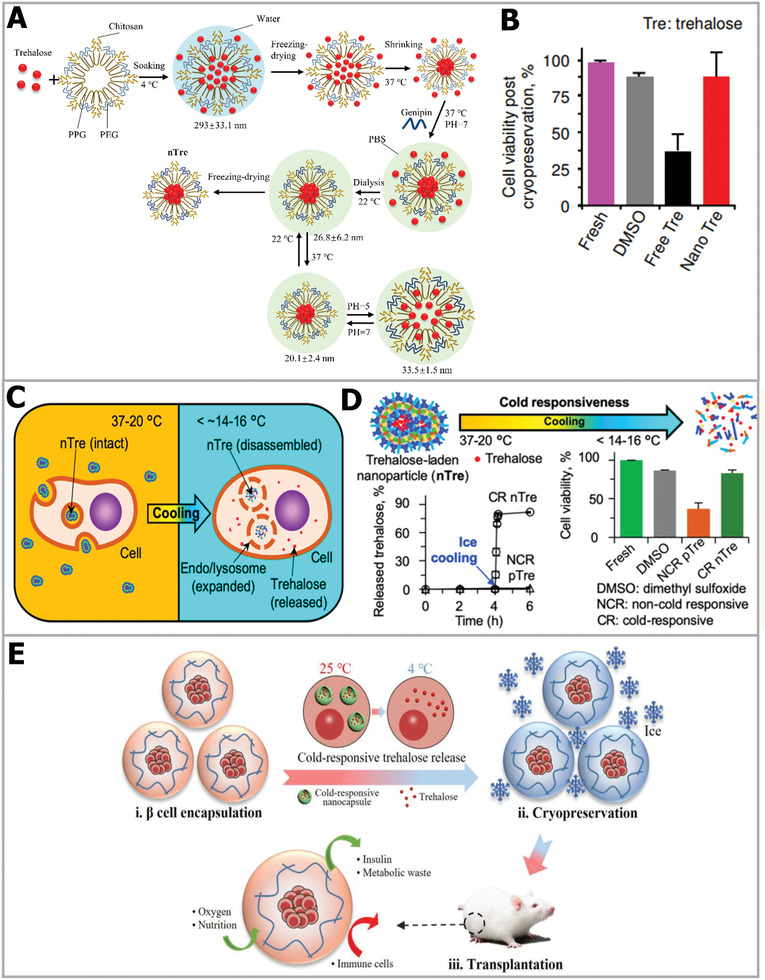
Trehalose‐delivery technology via responsive nanoencapsulation materials. A) The schematic illustration of encapsulating trehalose in pH‐responsive nanoparticles. B) Comparison of cell viabilities using different CPA treatments. A,B) Reproduced with permission.^[^ ^153^ ^]^ Copyright 2015, American Chemical Society. C) Schematic illustration of trehalose release. D) Cold‐responsive nanoparticles enhanced the cryopreservation efficiency. C,D) Reproduced with permission.^[^ ^161^ ^]^ Copyright 2019, American Chemical Society. E) Cold‐responsive nanoparticles and hydrogel synergistically reduced ice injury and facilitated the cryopreservation of *β* cells, and the recovered cells were transplanted for treatment. Reproduced with permission.^[^ ^162^ ^]^ Copyright 2019, Wiley‐VCH.

Responsive nanoparticles provide faster and more efficient approaches for cellular trehalose uptake. For instance, as depicted in **Figure** **12**A, Rao et al. designed and synthesized a kind of pH‐responsive genipin‐crosslinked Pluronic F12‐chitosan nanoparticles to encapsulate trehalose.^[^ ^153^ ^]^ After the cellular uptake of nanoparticles containing trehalose, trehalose can be released at pH 5 owing to the acid condition of late endosomes and lysosomes in mammalian cells. Based on this method, the cryopreservation efficiency after adding the pH‐responsive nanoparticles showed a comparable effect to DMSO (Figure 12B), demonstrating that intracellular trehalose effectively suppressed ice injury and facilitated the recovery of cells postcryopreservation. In order to adapt to the cooling process, Zhang et al. synthesized cold‐responsive nanoparticles that can release trehalose after exposure to cold temperature.^[^ ^161^ ^]^ The design of the cold‐responsive nanoparticles was mainly based on the transition of poly (*N*‐isopropylacrylamide‐*co*‐butyl acrylate) from hydrophobic to hydrophilic. As depicted in Figure 12C, the cold‐responsive nanoparticles can realize trehalose delivery below 20 °C, which is suitable for the cooling process for cryopreservation. Meanwhile, the fast and controlled trehalose release enabled comparable cryopreservation survival rates when DMSO was used, as shown in Figure 12D, implying the potential of trehalose delivery triggered by cold temperature for organic‐solvent‐free cryopreservation. Later, Cheng et al. further combined the cold‐responsive trehalose‐delivery strategy induced by cold temperature with an antifreezing hydrogel, thus synergistically achieving high‐efficiency DMSO‐free cryopreservation of islet *β* cells.^[^ ^162^ ^]^ As schematically illustrated in Figure 12E, this approach not only simultaneously reduced the ice damage to *β* cells, but also the thawed cells still retained blood glucose regulation function and could be transplanted in vivo for diabetes treatment. After transplantation of the cryopreserved *β* cells into the muscles of the legs, the blood glucose level of diabetic rats can recover the normal range and maintain for two weeks, indicating the functional integrity of *β* cells after undergoing cryopreservation. In brief, flourishing trehalose‐delivery biotechnology can reduce intracellular ice injury and offers a reliable approach for organic‐solvent‐free cryopreservation.

### 4.2 Cell Encapsulation for Extracellular Ice Inhibition

**Figure 13:**
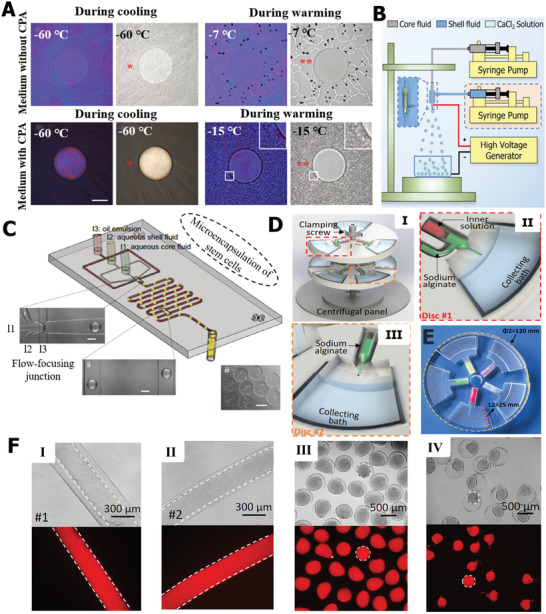
Extracellular ice inhibition based on cellular encapsulation strategy. A) The devitrification inhibition in the warming process. Reproduced with permission.^[^ ^163^ ^]^ Copyright 2015, Wiley‐VCH. B) The illustration of electrostatic spray. Reproduced with permission.^[^ ^146^ ^]^ Copyright 2018, Royal Society of Chemistry. C) The schematic diagram of microfluid. Reproduced with permission.^[^ ^163^ ^]^ Copyright 2015, Wiley‐VCH. D) Cell encapsulation based on centrifugal microfluids. E) The simple centrifugal microfluid device. F) The fabrication of single microcapsules and fibers and double‐layer structure. D–F) Reproduced with permission.^[^ ^166^ ^]^ Copyright 2015, Elsevier.

Extracellular ice formation and growth is a crucial factor for cell survival in the cryopreservation process. To inhibit extracellular ice and reduce its cryodamage, researchers use biocompatible hydrogels to enclose living cells in capsules, and the protective structure outside the cell can effectively suppress extracellular ice. Especially for the vitrification cryopreservation method, it has been discovered that the cell encapsulation strategy using hydrogel can facilitate the vitrification inside capsules and inhibit the occurrence of devitrification in the process of thawing. More importantly, the 3D microenvironment of the encapsulated hydrogel is similar to the extracellular matrix and allows the diffusion of nutriments and waste products of cells, indicating that the cell‐laden microcapsules, based on encapsulation technology can be regarded as an entirety to cryopreserve, and for further applications such as in vivo transplantation and drug carriers.^[^ ^140^ ^]^ Cell encapsulation methods induce the formation of cell‐laden microcapsules and greatly affect the final cryopreservation efficiency. As shown in **Figure** **13**A, in the process of cryopreservation, the cell encapsulation strategy not only offers a protective barrier for cells to reduce extracellular ice damage, but also prevents ice nucleation propagation into cells.^[^ ^163^ ^]^ In general, cell encapsulation approaches can be roughly classified into the microfluid, extrusion, and emulsion methods.^[^ ^154^ ^]^ It is difficult to cover all the cell encapsulation methods in the application of cell cryopreservation; here, we present several typical examples to highlight the role of cell encapsulation in inhibiting ice for cell cryopreservation.

Extrusion methods are commonly used to fabricate controlled‐sized capsules that effectively suppress devitrification.^[^ ^212^ ^]^ Among extrusion methods, the electrostatic spray technology is typically utilized to produce high‐throughput microcapsules. As shown in Figure 13B, Zhang et al. fabricated cell‐laden alginate hydrogel microcapsules based on the electrostatic spray system.^[^ ^146^ ^]^ The crosslinked alginate hydrogel network can restrict the movement of water molecules and, thus, inhibit the devitrification. Interestingly, the method can also produce double‐layer microcapsules via tube‐in‐tube devices. The generation of hydrogel microcapsules based on microfluid technology is another typical method.^[^ ^33^, ^164^, ^165^ ^]^ He and co‐workers designed a microfluid device to fabricate cell–alginate hydrogel constructs,^[^ ^163^ ^]^ as described in Figure 13C. Under the shear force, the cells were encapsulated into the alginate, and the formed microcapsule constructs inhibited the development and growth of ice. It is worth noting that the hydrogel microencapsulation induced low‐CPA vitrification, which greatly decreased the toxicity caused by high‐CPA vitrification. More recently, Zhao's group proposed an innovative and simple centrifugal microfluid method to effectively generate cell‐laden constructs with controlled size and structure.^[^ ^166^ ^]^ As schematically illustrated in Figure 13D, tunable microcapsules were produced via the centrifugal jetting disc. The simple and cheap device based on centrifugal microfluid (Figure 13E) was able to fabricate multitunable cell‐laden constructs, such as core/shell structures and simple capsules and fibers (Figure 13F). Cell encapsulation can also achieve large‐volume cryopreservation, and the post‐thaw cell‐laden microcapsules can be assembled and applied for cell‐based therapy.^[^ ^167^ ^]^ Moreover, cell encapsulation based on the drop cell printing approach has promising potential for ice inhibition in cryopreservation.^[^ ^168^, ^169^, ^170^ ^]^ Briefly, cell encapsulation is an effective engineering strategy to inhibit the formation and propagation of ice in both cooling and warming procedures, reducing cryoinjury to cells.

### 4.3 Bioinspired Structure Design for Ice Inhibition

In nature, excellent biological organisms are evolving, leading to different morphological and structural functions to adapt and expand in incompatible environments. One of the optimized properties is the unique biological structure that suppresses ice and reduces cryoinjury. For example, the natural cytoderm structure provides a “protective coat” for plant cells, which can avoid the ice crystal directly contacting the organelle and ice nucleation from expanding into the cell. Similarly, some mammalian cells also possess special structural features to protect them from external physical damage. Hence, natural organism structures have offered an important source of inspiration for designing a bioinspired anti‐icing strategy.

**Figure 14:**
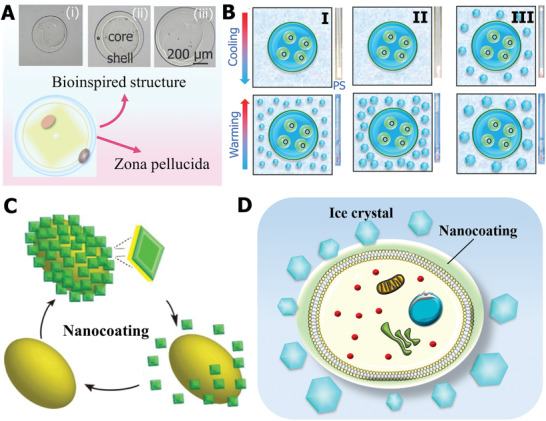
Ice inhibition by bioinspired structure design. A) Core–shell structure design bioinspired by the zona pellucida of egg cells. B) Proposed ice‐inhibition mechanism of bioinspired structure. A,B) Reproduced with permission.^[^ ^147^ ^]^ Copyright 2017, Wiley‐VCH. C) Nanocoating design bioinspired by cell wall structure. Reproduced with permission.^[^ ^46^ ^]^ Copyright 2017, Wiley‐VCH. D) Possible protection mechanism of nanocoating during cryogenic process.

Among various mammalian cells, the zona pellucida structure of egg cells provides a natural “protective coat” for them. Inspired by the zona pellucida structure, core–shell structure cell–biomaterial constructs were designed as displayed in **Figure** **14**A. The cell constructs with a tunable core–shell structure not only facilitated the vitrification of the low‐CPA part but also inhibited the ice injury both in the cooling and thawing processes.^[^ ^147^ ^]^ Combined with different CPA formulas, the bioinspired core–shell structure showed an excellent protective effect on cryopreserved cells. The mechanism underlying the prevention of ice formation and growth can be described as schematically illustrated in Figure 14B. For the vitrification cryopreservation method using different CPA formulas, the devitrification and ice recrystallization during the warming process occur (Figure 14B‐I,II), and the bioinspired core–shell structure prevented ice recrystallization propagating into cells and causing mechanical damage. It is likely that, although partial vitrification probably formed large ice crystals outside the core–shell structure during the cooling stage and the devitrification/ice recrystallization also occurred during the warming process (Figure 14B‐III), the core–shell structure can prevent direct contact with ice. Moreover, it is worth noting that the core–shell was regarded as a barrier to prevent ice nucleation from propagating into the cell.

Bioinspired by the structure of the cytoderm, it is desirable to design an artificial coating on the surface of cells. In the past few years, a new class of biocompatible nanoparticles has been fabricated, such as silica, titania, graphene, and polydopamine, the function of which is to coat cell membranes.^[^ ^171^, ^172^ ^]^ Therefore, nanocoating provides a promising method for developing artificial cytoderm structures. As shown in Figure 14C, the nanocoating can effectively modify the surface of cell membranes, which endowed the mammalian cells with an artificial protective coating.^[^ ^46^ ^]^ During the cryopreservation process, the nanocoating not only modulated the interfacial energy between the ice and cell membrane but also resisted the ice propagation into the cell and stopped the permeation of small ice crystals (Figure 14D). In addition, different shell strengths can be achieved by preparing nanocoatings with different thicknesses. It is also worth noting that the nanocoating should have outstanding biodegradability, which is crucial for the further application of cells postcryopreservation. With the rapid development of bioinspired science, it is reasonable to believe that more natural structures will be mimicked to develop anti‐icing strategies for cell cryopreservation.

## 5 Ice Inhibition Based on External Physical Field

Great progress and advances have been made in regulating ice‐crystal formation and suppressing ice‐crystal growth based on external physical field technologies. Presently, the ice crystals in cryopreservation are usually subjected to two effects within the external physical field: 1) the physical field can provide sufficient warming rates, thereby inhibiting the occurrence of recrystallization/devitrification and 2) the external applied physical field is able to influence the movement state of water molecules and further modify the formation and growth of ice in the process of cooling. As briefly summarized in **Table** **2**, ice nucleation and growth regulation based on the external physical field has attracted widespread attention.^[^ ^173^ ^]^ In this section, we emphasize the ice‐modulation effects based on the physical field technology, and how it improves the cryopreservation of cells, tissues, and organs.

**Table 2:** Comparison of influence effects of different physical fields on ice

Physical fields	Ice influence effects	Intrinsic mechanism	Refs.
Magnetic field	Increases supercooling degree Delays ice nucleation	Affects water molecule movement Electromagnetic heating effect	^[^ ^174^ ^]^
	Reduces ice‐grain size Uniform ice melting	Influences dipole polarization of water molecules	
Static electronic field	Induces ice nucleation Decreases ice‐grain area Increased phase transition time	Increases nucleation probability Changes free energy	^[^ ^175^ ^]^
Alternating electronic field	Inhibits ice nucleation Influences nucleation temperature	Suppresses water molecule aggregation	^[^ ^176^ ^]^
Sound field	Induces ice nucleation Regulates ice shape and size Enhances heat transform	Bubble and microflow formation Breaks large ice crystals	^[^ ^179^ ^]^
Laser field	Fast ice melting	Photothermal‐transformation effect	^[^ ^178^ ^]^

### 5.1 Magnetic Field

**Figure 15:**
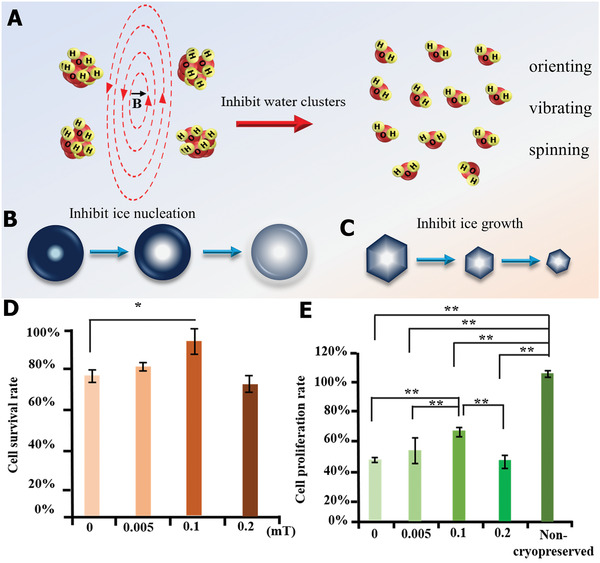
Ice modulation via external magnetic field in the freezing process. A) Possible ice‐inhibition mechanism of magnetic field via suppressing water clusters. B) Ice nucleation inhibition based on magnetic field. C) The ice‐crystal growth inhibition under magnetic field. D) The cell survival rates under magnetic fields of different intensities. E) Comparison of cell proliferation rate postcryopreservation in magnetic fields of different intensities. D,E) Reproduced with permission.^[^ ^182^ ^]^ Copyright 2013, Elsevier.

Currently, magnetic freezing technology has been proven to prevent cell destruction by regulating ice crystals and the supercooling degree of the solution.^[^ ^174^ ^]^ The external magnetic field can affect the movement state of water molecules by orienting, vibrating, and spinning, thereby inhibiting the cluster to some extent and promoting supercooling (**Figure** **15**A). Microscopically, the hydrogen nuclei possess a net magnetic moment; thus, the applied magnetic field will lead their motion around the direction of the magnetic field.^[^ ^179^ ^]^ Meanwhile, the external magnetic field can inhibit ice nucleation, resulting in uniform and tiny ice crystals, owing to the direct influence on the freezing kinetics of water, as shown in Figure 15B,C. Moreover, it has been reported that the magnetic field also affects the hydrogen bonds between water molecules, thus decreasing water clusters.^[^ ^174^, ^180^ ^]^ Notably, the underlying mechanism of the magnetic field's effect on ice in the freezing process can be elucidated by quantum electrodynamics, but it is beyond the scope of this review. Thus, we mainly concentrate on the magnetic field's influence on ice and its application in the field of cell cryopreservation. In the application of cryopreservation, Iwasaka et al. discovered that the magnetic field can prohibit ice formation during freezing.^[^ ^181^ ^]^ Moreover, it was also found that variations in the applied magnetic field can prevent intracellular ice. Kojima et al. successfully enhanced the efficiency of cryopreserving mesenchymal stem cells (MSCs) based on the magnetic field.^[^ ^182^ ^]^ As shown in Figure 15D, the survival rate of MSCs was higher when there was a magnetic field intensity of 0.1 mT than that without the magnetic field. However, the cells’ recovered viability slightly decreased once the intensity increased to 0.2 mT, which indicated that a stronger alternating magnetic field probably caused mechanical injury to cells. At the same time, the cell proliferation rate showed a consistent trend with cell viabilities (Figure 15E). More importantly, it has been proven that the external magnetic field had no side effect on the differentiation ability and genetic material of stem cells, demonstrating the safety and reliability of the magnetic field during freezing.^[^ ^183^ ^]^

**Figure 16:**
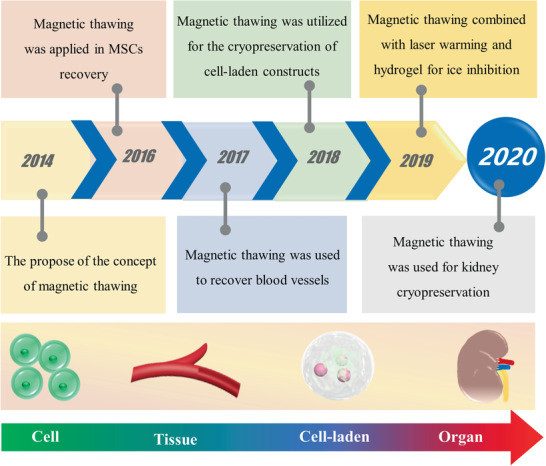
The important development history node diagram of magnetic thawing and its applications for cryopreservation from cell to organ scale.

In addition to the freezing process, the external magnetic field remarkably enhanced the thawing efficiency for the recovery of cryogenic cells. It is well known that the warming rate is a significant factor in the survival of cryopreserved biological samples.^[^ ^25^ ^]^ If the warming rate is not rapid enough, the occurrence of recrystallization/devitrification will cause fatal cryodamage to cells. Hence, the key point is how to achieve fast and uniform recovery during the thawing process. Magnetic iron‐oxide nanoparticles such as Fe_3_O_4_ can rapidly convert external magnetic energy into thermal energy, which offers an effective solution to solve the ice‐recrystallization problem. Although the proposed concept of magnetic thawing is relatively recent, as shown in **Figure** **16**, rapid progress has been made in cryopreservation, from the cellular to the organ scale, based on magnetic thermal thawing technology.

**Figure 17:**
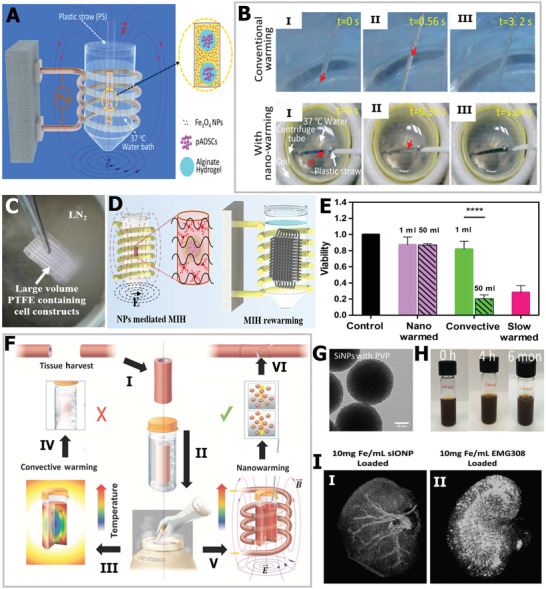
IRI or devitrification inhibition based on magnetic thawing. A) The schematic illustration of magnetic warming. B) Comparison of devitrification under conventional water bath warming and magnetic thawing. A,B) Reproduced with permission.^[^ ^185^ ^]^ Copyright 2018, American Chemical Society. C) The cooling of 3D PTFE containing cells. D) Magnetic warming for large‐volume PTFE. C,D) Reproduced with permission.^[^ ^186^ ^]^ Copyright 2018, Wiley‐VCH. E) Quantitative viability comparison. F) The cryopreservation of blood vessel tissue using magnetic field. E,F) Reproduced with permission.^[^ ^187^ ^]^ Copyright 2017, American Association for the Advancement of Science. G) TEM image of nanoparticles with modification. H) The dispersibility and stability of prepared nanoparticles. I) Micro‐CT 3D photograph of kidneys perfused nanoparticles I) with and II) without modification. G–I) Reproduced with permission.^[^ ^188^ ^]^ Copyright 2020, Wiley‐VCH.

Zhao and co‐workers first utilized a magnetic field to realize the rewarming of human umbilical cord matrix MSCs.^[^ ^184^ ^]^ Compared with untreated cells, the viability of thawed cells can be remarkably increased. In a later study, they successfully combined magnetic warming with hydrogel encapsulation, as illustrated in **Figure** **17**A, achieving low‐CPA vitrification cryopreservation of stem cells.^[^ ^185^ ^]^ The underlying mechanism of IRI in the warming process was investigated. As depicted in Figure 17B, the devitrification ended at 0.36 and 0.56 s, and ice crystals completely melted at 1.84 and 3.2 s for the magnetic warming and traditional water bath, respectively. Therefore, it can be concluded that the accelerated thawing induced by the magnetic field resulted in IRI, thus reducing cryoinjury to cells. Furthermore, another advantage of magnetic warming is the uniform space heating effect, which has great potential for large‐scale cell and organ cryopreservation. For example, Cao et al. designed scalable polytetrafluorethylene (PTFE) equipment through 3D printing, to contain cells for massive‐volume cryopreservation (Figure 17C).^[^ ^186^ ^]^ During the thawing procedure, the magnetic field was capable of providing rapid and uniform warming for the large‐volume PTFE cell container (Figure 17D), and decreased the ice damage triggered by the recrystallization/devitrification.

It is more difficult to cryopreserve tissue, owing to its larger volume and more complex physiobiological structure. In particular, ice recrystallization can cause thermal–mechanical stress and cracks to cryopreserved tissues. In order to address this issue, Bischof and co‐workers successfully realized high‐efficiency cryopreservation of blood vessels based on a magnetic warming strategy.^[^ ^187^ ^]^ As shown in Figure 17E, magnetic thawing increased the viability of porcine carotid both in 1 mL (solid purple and green) and 50 mL (patterned purple and green) systems, which was apparently higher than conventional slow warming and convective warming. As shown in Figure 17F, traditional convective heating probably led to blood vessel crack and failure, while the alternating magnetic field was able to endow the blood vessel with uniform heating, and the uniformly distributed magnetic nanoparticles could melt the ice crystals rapidly. More impressively, they synthesized silica‐coated iron‐oxide nanoparticles, as shown in Figure 17G, and the prepared nanoparticles possessed excellent biocompatibility, stability, dispersibility, and high heating effects (Figure 17H).^[^ ^188^ ^]^ They successfully perfused into and washed out the nanoparticles from a rat kidney, as displayed in Figure 17I‐I, which offered a promising approach for future crucial organ cryopreservation. However, the uncoated nanoparticles would aggregate inside the kidney, and thus, not achieve form warming (Figure 17I‐II). In summary, magnetic field technology is an effective strategy to inhibit the formation and growth of ice crystals, ensuring multiscale biological sample cryopreservation.

### 5.2 Laser Field

**Figure 18:**
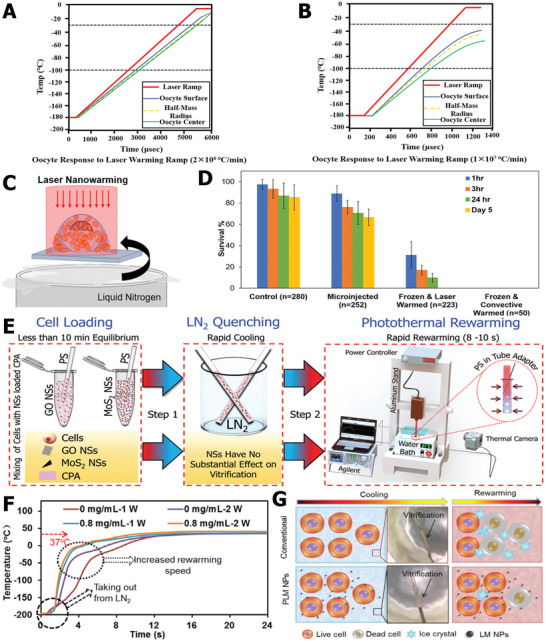
Laser thawing suppressed ice inhibition and devitrification for cryopreservation. A) The response curves of oocytes induced by laser warming at 2 × 10^6^ °C min^−1^ and B) 1 × 10^7^ °C min^−1^. A,B) Reproduced with permission.^[^ ^194^ ^]^ Copyright 2014, Elsevier. C) The diagram of laser warming. D) Comparison of survival viabilities of embryos using four treatments. C,D) Reproduced with permission.^[^ ^195^ ^]^ Copyright 2017, American Chemical Society. E) The cryopreservation procedures based on laser warming. Reproduced with permission.^[^ ^178^ ^]^ Copyright 2018, Royal Society of Chemistry. F) The temperature trend of vitrification solution with and without liquid nanoparticles in warming process. G) The schematic illustration of liquid nanoparticles inhibiting devitrification in warming process. F,G) Reproduced with permission.^[^ ^196^ ^]^ Copyright 2020, Elsevier.

In recent years, the flourishing development of nanoparticles with excellent photothermal conversion properties has been widely applied in the field of biomedical engineering, including cancer treatment, drug release, and biological imaging.^[^ ^189^, ^190^, ^191^ ^]^ Nanoparticles with photothermal‐transformation effects combined with near‐infrared laser irradiation provide an ultrarapid heating platform and have great potential to restrict ice recrystallization or devitrification in the warming process for millimeter‐scale cryopreservation.^[^ ^38^, ^192^ ^]^ Jin and Mazur first utilized laser fields to inhibit ice recrystallization during the warming process, improving the cryopreservation efficiency of oocytes.^[^ ^193^ ^]^ Previous studies have proved that a rapid warming rate is critical to reduce ice injury. Based on this, they analyzed the distribution of temperature under a laser field.^[^ ^194^ ^]^ As shown in **Figure** **18**A,B, the calculation results indicated that the laser‐induced heat can rapidly and evenly recover oocytes. Subsequently, Khosla et al. used gold nanorods (GNRs) to increase the heating rates in the cryopreservation of embryos.^[^ ^195^ ^]^ Because of the sufficiently biocompatible GNRs, they successfully injected these GNRs into embryos. As demonstrated in Figure 18C, the laser produced rapid and uniform warming rate both inside and outside the embryo, to prevent ice formation. Based on this laser‐field thawing technology, the banking efficiency of embryos was significantly increased compared with traditional convective warming. The GNR‐assisted embryo warming presented survival rates of 31%, 17%, and 10% after 1, 3, and 24 h, respectively, but the viabilities of the control group thawed by convective heating were 0% (Figure 18D).

The key point of fast warming is dependent on the photothermal‐transformation efficiency of nanoparticles. In a recent study, Panhwar et al. utilized 2D nanosheets (GO and MoS_2_) to inhibit ice growth and enhance the warming rates.^[^ ^178^ ^]^ As shown in Figure 18E, the CPA solution containing GO or MoS_2_ nanosheets and cell samples was directly embedded into liquid nitrogen; subsequently, the laser generated sufficient heating effects to warm the biological samples. Ultimately, the minimum concentration of 0.02% GO improved the final cryopreservation efficiency. Notably, the distribution of GO and MoS_2_ in the solution is crucial to melt ice crystals uniformly, and to determine the homogeneity of thawing. Hence, it is important to avoid the aggregation of nanomaterials. More recently, Rao and co‐workers proposed laser‐triggered warming to suppress vitrification using soft liquid metal nanoparticles.^[^ ^196^ ^]^ The nanoparticles showed a higher photothermal conversion efficiency of up to 52%, thus achieving a faster warming rate to reduce ice cryoinjury (Figure 18F). The laser's mechanism of ice regulation is shown in Figure 18G. For conventional water bath rewarming, visible vitrification would occur and accompany growing ice crystals, resulting in fatal damage to cryopreserved cells, whereas laser‐field recovery technology can effectively inhibit and outrun the formation and growth of ice. The procedure and device using laser warming is simple, but the limitation of laser warming is that it can only be applied in small‐scale sample cryopreservation. Hence, combining the laser field with other physical fields may provide an ultrarapid and uniform warming rate to suppress ice recrystallization or devitrification.

### 5.3 Electric Field

**Figure 19:**
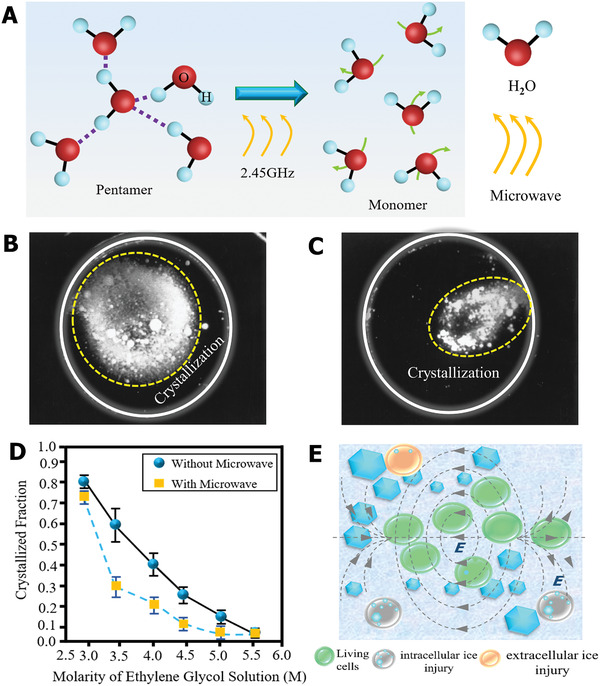
Effects of electric field on ice‐crystal formation and growth. A) Schematic illustration of disturbing effect of microwave irradiation on water pentamer. Reproduced with permission.^[^ ^198^ ^]^ Copyright 1992, Wiley‐VCH. Image of ice crystallization B) without microwave irradiation and C) with microwave irradiation. D) Quantitative analysis of visible ice crystallization fraction under different CPA concentrations with and without microwave irradiation. B–D) Reproduced with permission.^[^ ^200^ ^]^ Copyright 1997, Elsevier. E) Schematic diagram showing that extracellular and intracellular ice inhibition facilitated the survival of cryopreserved cells.

An external alternating electric field can significantly influence ice formation. Microwaves, as a typical oscillating electric field, have been shown to inhibit ice nucleation and crystallization.^[^ ^197^ ^]^ The underlying mechanism of the microwave radiation effect can be ascribed to the interaction between the oscillating electric field and dipolar water molecules, which suppresses ice nucleation and crystallization. It is well known that intermolecular forces such as hydrogen bonds exist between water molecules. In terms of cluster theory, formed hydrogen bonds lead to the occurrence of a crystal‐like cluster when the temperature decreases. Further, the ice nucleation and growth behavior are also closely related to the water molecule cluster. As shown in **Figure** **19**A, under microwave irradiation, the oscillating electric field can disturb the equilibrium relationship of the cluster by applying a torque on water molecules.^[^ ^198^ ^]^ The applied torque results in an increased number of isomeric configurations, consequently decreasing the probability of the crystal lattice.^[^ ^199^ ^]^ Notably, the kinetic process of water molecules greatly depends on the microwave parameters, such as frequency and power. To understand the effect of microwave radiation on the freezing process, the mechanism of ice inhibition from the viewpoint of molecular dynamics was elucidated. Similarly, it was discovered that dipole polarization of water molecules rotated once the water molecules were subjected to microwaves, altering the aggregation state of water molecules and breaking the hydrogen bonds between them. Therefore, the alternating electronic field increased the energy barrier for the water molecules to transform into ice, thereby inhibiting ice growth during freezing.

Based on this physical and noncontact ice‐regulation strategy, microwaves have been used for assisting biological cryopreservation. In 1992, Hanyu et al. first applied microwaves for the cryopreservation of small tissue blocks, and described the back mechanism of increasing cryopreservation quality.^[^ ^198^ ^]^ After that, Gao and co‐workers studied the interaction effect between microwaves and ethylene glycol (CPA) on solution crystallization.^[^ ^200^ ^]^ They discovered that the occurrence of microwave radiation can apparently reduce the fraction of ice crystallization under the same CPA concentration. As shown in Figure 19B,C, the amount of light diffraction without microwave irradiation was more remarkable than that with microwave radiation. Moreover, based on the quantitative assessment of the crystallization fraction, as shown in Figure 19D, the microwave reduced the average crystallization fraction by almost 56% in 3.5 m CPA and decreased ice formation by 66% in 4.5 m CPA. Hence, microwave radiation can reduce extracellular and intracellular ice damage during the freezing process and may be a potential method to promote cryopreservation efficiency and quality. In addition to the freezing process, microwaves with higher intensity also play an important role in suppressing devitrification and recrystallization during warming, and have been used for cryopreservation in the rewarming process since the 1970s.^[^ ^201^ ^]^ When the liquid water absorbs the microwave energy, which results in the occurrence of water molecule friction and increases the temperature of the system. Figure 19E illustrates the possible influence of microwaves on thawed cells, and the external microwave radiation can reduce the intracellular and extracellular ice recrystallization. Thus far, the major limitation of ice regulation based on microwave radiation might be how to overcome the radiation nonuniformity for the cryopreservation of large‐scale biological samples.

### 5.4 Sound Field

The propagation of sound waves can generate various physical and mechanical effects on the process of the formation and growth of ice crystals, which is also applied in tuning ice formation and ice size. Power ultrasound with a low frequency (20–100 kHz) and high intensity (>1 W cm^−2^) has been used to assist freezing and control ice crystals.^[^ ^177^ ^]^ Similar to other physical field approaches, ultrasound radiation is able to reduce ice size during freezing and enhance heat and mass transfer for cryogenic media, which is important for cell cryopreservation.

**Figure 20:**
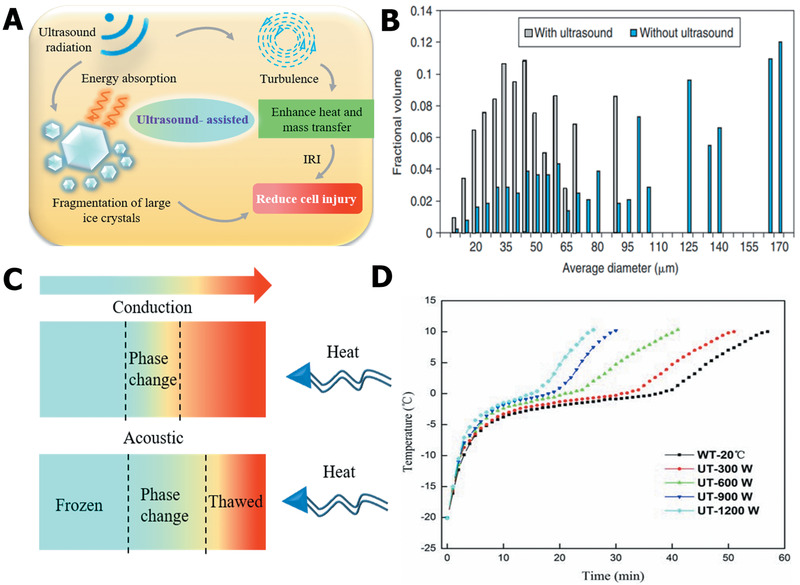
Ice regulation via external sound field. A) Schematic diagram of ice tuning of ultrasound field. B) Ultrasound effects on ice‐crystal size distribution. Reproduced with permission.^[^ ^177^ ^]^ Copyright 2005, Elsevier. C) Comparison of the effects of ultrasonic thawing and conventional thawing on phase transformation. Reproduced with permission.^[^ ^203^ ^]^ Copyright 2011, Elsevier. D) Temperature curves of thawing assisted by ultrasound fields with different powers. Reproduced with permission.^[^ ^205^ ^]^ Copyright 2014, Springer Nature.

As illustrated in **Figure** **20**A, when the solution is subjected to the ultrasound radiation effect, the ultrasonic energy can induce the fragmentation of large ice crystals. At the same time, the ultrasound field causes microstreaming, which significantly increases the efficiency of heat transfer, thereby shortening the duration time between the onset of ice crystallization and complete formation and reducing cellular damage. With respect to ice size distribution, Acton and Morris quantitatively analyzed the influence of ultrasound radiation on the freezing process of sucrose solution.^[^ ^202^ ^]^ As shown in Figure 20B, they found that ≈77% of ice crystals were within a diameter of 50 µm under ultrasound radiation; without ultrasound, the amount was 32%, indicating that the ultrasound field can lead to a smaller ice size distribution. Furthermore, ultrasound thawing technology also plays an important role in inhibiting ice growth. Generally, ultrasonic energy can be utilized to accelerate the phase transformation at the interface between cryogenic samples and ice. Compared with the traditional heat conduction method, absorbing ultrasound energy can promote the increase of water and generate high‐speed jets, which improves the heat transfer rate and induces faster ice/water phase transformation (Figure 20C).^[^ ^203^ ^]^ Currently, ultrasound thawing technology has been successfully applied in the field of food.^[^ ^204^ ^]^ Ultrasound with more power can shorten thawing time and further improve the quality of cryopreserved samples (Figure 20D).^[^ ^205^ ^]^ This method also has promising potential in cell recovery, and reduces ice‐recrystallization damage. It is worth noting that the appropriate parameters of ultrasound, such as radiation duration, intensity, and frequency, are crucial for regulating ice and biological cells. Excessive ultrasound energy might cause mechanical damage to the cell wall and other cell structures. Generally, the underlying ice‐regulation mechanisms of the ultrasonic field are not fully understood; the application of sound waves in the cryopreservation field is in the early stage, and more fundamental research and efforts are needed to establish the relationship between ice regulation and ultrasound field radiation.

## 6 Conclusion and Perspectives

As highlighted in this review, innovative materials and strategies for ice inhibition in cryopreservation have made significant progress. We first discussed the fundamental ice cryodamage mechanisms in the cooling–thawing process of cryopreservation. We also summarized current state‐of‐the‐art chemical ice‐inhibition molecules relying on the basic understanding of ice injury, that is, traditional CPAs, AFPs, synthetic polymers, nanomaterials, and hydrogels. Moreover, advanced engineering technologies for ice inhibition, including trehalose delivery, cell encapsulation, and bioinspired structure design, have been emphasized. Ultimately, the control of the formation and growth control of ice crystals based on external physical field strategies was also systematically described. Overall, regardless of intracellular and extracellular ice inhibition, it is vital to achieve high‐efficiency cryopreservation to meet the urgent need for emerging fundamental scientific research and practical clinical applications.

With respect to ice‐suppression materials, continuous efforts should be made to explore molecule‐level mechanisms, especially in the molecular interaction between ice‐inhibition materials and water/ice molecules. AFP, as a typical ice inhibitor in nature, provides new inspiration and guidance for anti‐icing materials design. Until now, remarkable progress has been made in the unraveling of the basic mechanism of AFPs on ice nucleation, shaping, and ice recrystallization.^[^ ^39^, ^40^ ^]^ Despite these developments, some work remains challenging. First, a deep understanding of the relationships between the 3D structure of AFPs and the ability to tune the formation, growth, and shaping of ice still needs to be fully developed. Second, the universal explanation of natural AFPs for ice inhibition is controversial, and the quantitative analysis of ice inhibition, in theory, has not been established. These challenges will pull scientific researchers to explore a diverse range of bioinspired AFP molecules and/or polymers in structure or functional similarities for ice inhibition and further application in cryopreservation. It should be noted that the availability, cost, and awareness of toxicity are also crucial for assisting the cryostorage of biological samples.^[^ ^100^ ^]^

Advanced engineering strategies such as trehalose delivery, cell encapsulation, and bioinspired structure design have been described as methods to suppress ice and reduce chilling injury. Trehalose delivery can achieve intracellular ice inhibition as the sole nontoxic CPA. Cell encapsulation is capable of providing an integrated platform with ice control, long‐term storage, and cell‐based therapy functions based on cell constructs. Various structures in natural organisms offer a source of inspiration for ice suppression. Despite substantial progress, the main limitation of these methods is the low throughput at the laboratory scale. One of the future directions for investigation should focus on how to achieve macropreservation through these strategies to meet current clinic needs. Moreover, it is clear that cryopreservation single‐cell systems has been successful, whereas cryopreservation of entire organs remains a great challenge.^[^ ^206^ ^]^ Presently, the duration of organ cryopreservation after procurement greatly decreases the efficiency of organ transplantation and increases cost; therefore, successful cryopreservation of organs is essential for regenerative medicine. In general, cooling and warming rates are crucial factors for organ cryopreservation.^[^ ^207^ ^]^ Due to the complex extracellular architecture and vascular system, the formation and growth of ice will cause fatal injury to organs, destroying their integrity and function. The most promising method for organ cryopreservation might be vitrification, which avoids ice‐crystal formation and eliminates ice damage during the freezing process.^[^ ^208^, ^209^ ^]^ To address this issue, more nontoxic CPAs or other vitrification‐induced materials should be exploited. At the same time, a fast cooling rate is also significant, and can facilitate vitrification. In addition to physical and biological strategies, advanced engineering devices can be developed to accelerate the cooling process. For instance, a recent study reported an ultrarapid cooling approach based on superflash freezing.^[^ ^210^ ^]^ This method effectively vitrified the cells without adding any CPA, and achieved comparable cryopreservation efficiency to using conventional CPAs. Moreover, the flourishing development of microscopy, magnetic resonance imaging, and microfluid platforms will offer a new understanding of organ cryopreservation and facilitate further development.^[^ ^33^, ^204^ ^]^ Another important point is how to inhibit ice recrystallization and devitrification for large‐scale organs during thawing. Uniform and fast space warming based on an external physical field are effective heating methods, especially for magnetic warming. The direction of future development of warming is probably multiphysical and synergetic thawing. For example, the combination of magnetic and laser fields can achieve uniform and fast warming rates, which is essential to overcome the ice barrier. More importantly, the physical field provides both physically and biologically flexible warming, and cannot lead to structure and function variations of cryopreserved samples. In conclusion, with the rapid developments in chemistry, material synthesis, biochemistry, and engineering, we believe that advanced and flourishing cryopreservation will keep pace with current and emerging needs, bringing a bright future for regenerative medicine. We anticipate that this review of previous works will be helpful in promoting the development of safe, high‐quality, and high‐efficiency cryopreservation of biological samples.

## Conflict of Interest

The authors declare no conflict of interest.
